# Quantitative lung ultrasound detects dynamic changes in lung recruitment in the preterm lamb

**DOI:** 10.1038/s41390-022-02316-0

**Published:** 2022-09-27

**Authors:** Arun Sett, Gillian W. C. Foo, Kelly R. Kenna, Rebecca J. Sutton, Elizabeth J. Perkins, Magdy Sourial, Sheryle R. Rogerson, Brett J. Manley, Peter G. Davis, Prue M. Pereira-Fantini, David G. Tingay

**Affiliations:** 1 Neonatal Research, Murdoch Children’s Research Institute, Parkville, VIC Australia; 2 Newborn Research Centre, The Royal Women’s Hospital, Parkville, VIC Australia; 3 Joan Kirner Women’s and Children’s Hospital, Western Health, St Albans, VIC Australia; 4 Department of Obstetrics and Gynaecology, The University of Melbourne, Parkville, VIC Australia; 5 Paediatric Infant Perinatal Emergency Retrieval, The Royal Children’s Hospital, Parkville, VIC Australia; 6 Translational Research Unit, Murdoch Children’s Research Institute, Parkville, VIC Australia; 7 Department of Paediatrics, The University of Melbourne, Parkville, VIC Australia; 8 Department of Neonatology, The Royal Children’s Hospital, Parkville, VIC Australia

## Abstract

**Background:**

Lung ultrasound (LUS) may not detect small, dynamic changes in lung volume. Mean greyscale measurement using computer-assisted image analysis (Q-LUS_MGV_) may improve the precision of these measurements.

**Methods:**

Preterm lambs (*n* = 40) underwent LUS of the dependent or non-dependent lung during static pressure–volume curve mapping. Total and regional lung volumes were determined using the super-syringe technique and electrical impedance tomography. Q-LUS_MGV_ and gold standard measurements of lung volume were compared in 520 images.

**Results:**

Dependent Q-LUS_MGV_ moderately correlated with total lung volume (rho = 0.60, 95% CI 0.51–0.67) and fairly with right whole (rho = 0.39, 0.27–0.49), central (rho = 0.38, 0.27–0.48), ventral (rho = 0.41, 0.31–0.51) and dorsal regional lung volumes (rho = 0.32, 0.21–0.43). Non-dependent Q-LUS_MGV_ moderately correlated with total lung volume (rho = 0.57, 0.48–0.65) and fairly with right whole (rho = 0.43, 0.32–0.52), central (rho = 0.46, 0.35–0.55), ventral (rho = 0.36, 0.25–0.47) and dorsal lung volumes (rho = 0.36, 0.25–0.47). All correlation coefficients were statistically significant. Distinct inflation and deflation limbs, and sonographic pulmonary hysteresis occurred in 95% of lambs. The greatest changes in Q-LUS_MGV_ occurred at the opening and closing pressures.

**Conclusion:**

Q-LUS_MGV_ detected changes in total and regional lung volume and offers objective quantification of LUS images, and may improve bedside discrimination of real-time changes in lung volume.

**Impact:**

- Lung ultrasound (LUS) offers continuous, radiation-free imaging that may play a role in assessing lung recruitment but may not detect small changes in lung volume.
- Mean greyscale image analysis using computer-assisted quantitative LUS (Q-LUS_MGV_) moderately correlated with changes in total and regional lung volume.
- Q-LUS_MGV_ identified opening and closing pressure and pulmonary hysteresis in 95% of lambs.
- Computer-assisted image analysis may enhance LUS estimation of lung recruitment at the bedside.
- Future research should focus on improving precision prior to clinical translation.

## Introduction

Continuous imaging of lung recruitment in neonatal patients is an attractive alternative to indirect physiological estimators of lung volume, such as peripheral oxygen saturations and ventilation parameters, or conventional radiography.^1,2^ For this purpose, lung ultrasound (LUS) and electrical impedance tomography (EIT) are promising point of care, radiation-free imaging modalities.^3^ Ultrasound interaction with the pleura produces consistent artefact patterns that correspond with lung aeration,^4^ forming the foundations of visual scoring systems for LUS.^5,6^ Despite traditional LUS scoring systems reliably diagnosing some neonatal respiratory conditions,^5,7–9^ and detecting large lung volume changes in adults^10^ and in animal models of the preterm lung,^11^ they cannot measure small changes in lung volume.^12^ There are few reports of the accuracy of LUS against gold-standards measurements of lung volume such as gas washout, computed tomography (CT) or the super-syringe technique,^13^ and only one has specifically studied the preterm lung.^11^ Thus, whilst a role for LUS is established, some aspects such as the assessment of preterm lung recruitment need further evaluation.^14–16^

The shortcomings of traditional LUS may be overcome by supplementing interpretation with computer-assisted image analysis. Measurement of the average greyness of a whole or specific region of an image is a simple form of image analysis. Quantitative lung ultrasound with analysis of the mean greyscale value (Q-LUS_MGV_) measures the greyness of each pixel within a defined region of interest (ROI) and calculates a numerical mean grey value for the ROI. Though the human perception of greyscale colours is limited,^17^ Q-LUS_MGV_ is capable of resolving the full breadth of the greyscale spectrum,^13,18^ making it suitable for discriminating small changes in lung volume in real-time.

EIT measures the real-time change in lung volume by analysing changes in electrical bioimpedance associated with varying lung aeration.^3^ EIT has an established role in critical care research, and reliably maps the pressure–volume (PV) relationship of the preterm lung in humans and animals.^19–21^ As EIT measurement of lung recruitment has been validated against CT,^22,23^ it serves as an appropriate comparator for LUS validation studies.

We hypothesised that Q-LUS_MGV_ would detect real-time changes in lung volume, demonstrate pulmonary hysteresis and identify the opening and closing pressure of the lung. To explore this, we compared Q-LUS_MGV_ of the pleural region of LUS images acquired from preterm lambs during mapping of the PV relationship of the respiratory system to global and regional lung volumes measured by the super-syringe method^21^ and EIT respectively.

## Methods

This study was part of a larger programme investigating the role of different respiratory strategies in the initiation of preterm lung injury at birth. The study was approved by the Murdoch Children’s Research Institute Animal Ethics Committee, Melbourne, Australia in accordance with National Health and Medical Research Council (Australia) guidelines and is reported as per the ARRIVE guidelines.^24^

### Animal preparation

Preterm lambs of 124–128 days gestation (term gestation 143–145 days) were delivered from anaesthetised, betamethasone exposed, Border-Leicester/Merino ewes via caesarian section. Following exteriorisation for carotid vessel instrumentation, intubation proceeded with a 4.0 mm cuffed endotracheal tube (ETT) followed by passive lung liquid drainage. As part of the protocol of the primary study, after 15 min of mechanical ventilation, lambs were maintained on placental support for 30 min whilst apnoeic with the ETT clamped. Next, the ETT was unclamped and opened to the atmosphere for 2 min whilst lambs were apnoeic and the static PV relationship of the respiratory system was mapped using a calibrated glass syringe as previously described.^21^ Lungs were inflated and then deflated using pre-defined pressure increments between atmospheric (0 centimetres of water [cm H_2_O]) and maximal inflation pressure (35 cm H_2_O). At completion, the umbilical cord was clamped and a lethal dose of sodium pentobarbitone (100 mg/kg) was administered.

### Lung ultrasound (LUS)

LUS was performed using a Logiq E (GE Healthcare, Wauwatosa, WI) and Terason USMART 3200T (Terason, Burlington, MA) ultrasound system with a 12-megahertz broadband linear transducer. Gain was set to 50 decibels, depth to 2.5 cm and the focal zone was positioned at the pleural line. Filters were deactivated. Details on ultrasound settings and techniques are described in the Supplementary methods. All animals were imaged in the supine position. Ultrasound images were acquired from the right lower lateral (dependent) and right anterior upper (non-dependent) thorax at each pressure increment during the inflation and deflation series. These correspond to the central and ventral EIT regions respectively. Sonographic opening and closing pressure were defined as the pressure increment preceding the largest change in Q-LUS_MGV_. Sonographic hysteresis was defined as the presence of distinct inflation and deflation limbs on PV curves constructed from Q-LUS_MGV_, with on average higher measurements on the deflation limb when compared to the corresponding inflation pressures.

### Computer-assisted greyscale analysis

**Fig. 1:**
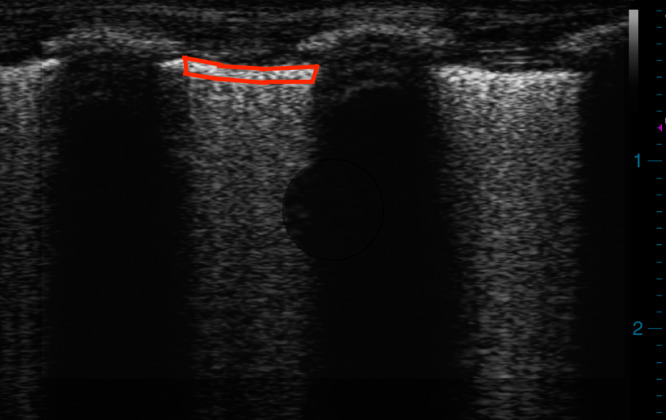
ROI (red tracing) for measurement of Q-LUS_MGV_. The ROI was delineated by (1) superior margin defined by the pleural surface; (2) lateral margin defined by the rib shadows; and (3) inferior margin defined by a depth of 50 pixels corresponding to one millimetre in depth. Q-LUS_MGV_; quantitative lung ultrasound mean grey value.

Uncompressed LUS images taken at each pressure increment were de-identified and imported into FIJI, ImageJ (National Institute of Health, Bethesda, Maryland).^25^ Representative still images were selected and analysed in 8-bit format for measurement of Q-LUS_MGV_ of the pleural ROI. Two investigators (A.S., 4-year LUS experience and G.F. 1-year LUS experience) manually delineated the pleural ROI (Fig. 1) on anonymized images in random order. Q-LUS_MGV_ was determined by measuring the sum grey value of all the pixels within the ROI and dividing the result by the total number of pixels within the ROI^25^ using the built-in measurement package. In 8-bit pixel depth, Q-LUS_MGV_ ranges from 0 (black) to 255 (white). Q-LUS_MGV_ is measured in arbitrary units.

### Electrical impedance tomography

Continuous EIT (Pioneer system, Sentec AG, Landquart, Switzerland) assessment of lung volumes was made at 48 frames/s from before commencing ventilation to study completion, including the mapping of the PV relationship, using our previously reported methodology.^21^ Data were reconstructed using an anatomically correct finite element model of the lamb thorax filtered to the respiratory domain.^3,20^ The time-course EIT signal for the whole lung was calibrated against the volume changes measured by the super syringe,^21^ and the lung volumes of the right whole, dorsal, central and ventral lung were determined from weighting the EIT pixel distribution of each region to the calibrated whole lung volumes.^26,27^

### Statistical analysis

As this study was part of a larger research programme, a convenience sample of 40 lambs was chosen. Baseline characteristics were reported as mean and standard deviation. Q-LUS_MGV_ and lung volumes were reported as median and interquartile range (IQR). Change in Q-LUS_MGV_ was defined as the increase in value relative to that measured at 0 cm H_2_O. Correlation was calculated using Spearman’s correlation coefficient (rho) as Q-LUS_MGV_ distribution was skewed. A strong correlation was defined as rho ≥0.70, moderate ≥0.50, fair ≥0.30 and weak <0.30. Friedman’s test and a post hoc Wilcoxon signed rank sum test with a Bonferroni correction for multiple comparisons was used to compare Q-LUS_MGV_ between each pressure increment. The pooled Q-LUS_MGV_ of the inflation and deflation series were fitted to the sigmoidal model of the PV relationship proposed by Venegas et al.^28^ using least-squares non-linear regression. The predicted opening and closing pressures were then calculated from the model by differentiating the regression equation using the best-fit parameters and finding peaks in the differential curve. This corresponded to the maximum rate of change in both Q-LUS_MGV_ and EIT curves. Interobserver variability was calculated using an intraclass correlation coefficient with a two-way random effects model. Significance was set at <0.05. Analysis was performed using GraphPad Prism (V9.1.2, GraphPad Software, San Diego) and R (R: A Language and Environment for Statistical Computing, Vienna, Austria, 2021).^29^

## Results

Forty lambs were studied (62% male). In our cohort, no pneumothoraces occurred during ventilation or mapping of PV relationship and no lambs had foetal distress or acidosis. A total of 520 LUS images were acquired (260 dependent and 260 non-dependent). Characteristics of the lambs are shown in Table 1.

**Table 1:** Lamb characteristics (*n* = 40).

Mean (SD) gestational age, days	125 (1)	
Male, *n* (%)	25 (62)	
Singleton, *n* (%)	12 (30)	
Mean (SD) birth weight, g	3002 (450)	
Arterial blood gas analysis, mean (SD)	Cord	15 min
pH	7.36 (0.05)	7.40 (0.06)
pCO_2_ (mm Hg)	44 (4)	36 (4)
Base excess (mmol/l)	−0.6 (2.9)	−2.0 (3.2)

*pCO*_*2*_ partial pressure of carbon dioxide, *SD* standard deviation.

**Fig. 2:**
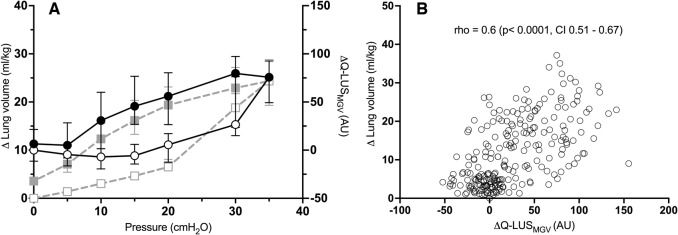
Dependent lung imaging. **A** Static PV curve derived from the super-syringe method (squares, grey dashed line) and ∆Q-LUS_MGV_ (circles, solid black line). All data are represented as median (IQR). ∆Q-LUS_MGV_ is normalised to baseline. Open shapes represent the inflation limb and closed shapes the deflation limb. **B** Correlation between ∆Q-LUS_MGV_ and total lung volume. AU arbitrary units, CI confidence interval, cm H_2_O centimetres of water, ml/kg, rho Spearman’s correlation coefficient, Q-LUS_MGV_ quantitative lung ultrasound mean grey value. Left axis: lung volume (ml/kg). Right axis: ∆Q-LUS_MGV_.

**Fig. 3:**
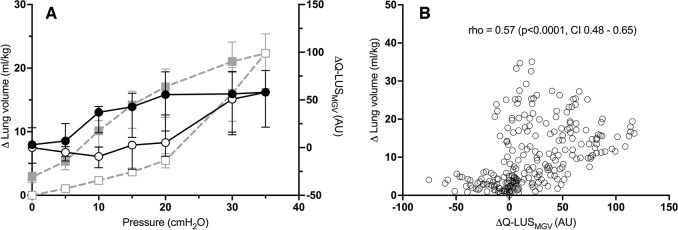
Non-dependent lung imaging. **A** Static PV curve derived from the super-syringe method (squares, grey dashed line) and ∆Q-LUS_MGV_ (circles, solid black line). All data are represented as median (IQR). ∆Q-LUS_MGV_ normalised to baseline. Open shapes represent the inflation limb and closed shapes the deflation limb.  **B** Correlation between ∆Q-LUS_MGV_ and total lung volume. AU arbitrary units, CI confidence interval, cm H_2_O centimetres of water, ml/kg, rho Spearman’s correlation co-efficient, Q-LUS_MGV_ quantitative lung ultrasound mean grey value. Left axis: lung volume (ml/kg). Right axis: ∆Q-LUS_MGV_.

Figures 2 and 3 show measured lung volumes at each pressure increment during mapping of the PV relationship (grey dashed line). The median (IQR) lung volume at 35 cm H_2_O was 24 (19–28) ml/kg (of body weight) and 22 (17–25) ml/kg in the dependent and non-dependent imaging groups, respectively. Hysteresis was evident in all lambs (individual PV relationships, Supplementary Figs. E1 and E2). Opening and closing pressures were 20 cm H_2_O and 10 cm H_2_O respectively in 19 out of 20 lambs in the dependent group and 18 out of 20 lambs in the non-dependent group. This corresponded to median (IQR) lung volumes of 6.5 (6–8) ml/kg and 12 (10–15.5) ml/kg in the dependent group and 5.5 (4.5–6.5) ml/kg and 10 (7.5–12) ml/kg in the non-dependent group.

### Total lung volume derived from the super-syringe method

#### Dependent LUS

Q-LUS_MGV_ of the dependent lung moderately correlated with changes in total lung volume (rho = 0.60, *p* < 0.0001, CI 0.51–0.67, Fig. 2). Q-LUS_MGV_ was able to differentiate between pressures (median volume [IQR]) of 15 cm H_2_O (5 [4–5] ml/kg) and 30 cm H_2_O (18 [15–22] ml/kg) during the inflation series (*p* = 0.01), and 20 cm H_2_O (19 [15–23] ml//kg) and 10 cm H_2_O (12 [10–16] ml/kg) during the deflation series (*p* = 0.04) (Supplementary Table E1). Q-LUS_MGV_ demonstrated distinct inflation and deflation limbs, and pulmonary hysteresis in all lambs (Individual pressure/Q-LUS_MGV_ relationships, Supplementary Fig. E3a, b).

#### Non-dependent LUS

Q-LUS_MGV_ of the non-dependent lung moderately correlated with changes in total lung volume (rho = 0.57, *p* < 0.0001, CI 0.48–0.65, Fig. 3). Q-LUS_MGV_ was able to differentiate between pressures (median volume [IQR]) of 20 cm H_2_O (5 [4–7] ml/kg) and 30 cm H_2_O (16 [12–20] ml/kg) during the inflation series (*p* = 0.01), and 10 cm H_2_O (10 [8–12] ml//kg) and 5 cm H_2_0 (5 [4–7] ml/kg) during the deflation series (*p* = 0.01) (Supplementary Table E2). Q-LUS_MGV_ demonstrated distinct inflation and deflation limbs, and pulmonary hysteresis in 18/20 lambs (individual pressure/Q-LUS_MGV_ relationships, Supplementary Fig. E4a, b).

### Regional lung volume derived from EIT

#### Dependent LUS

**Fig. 4:**
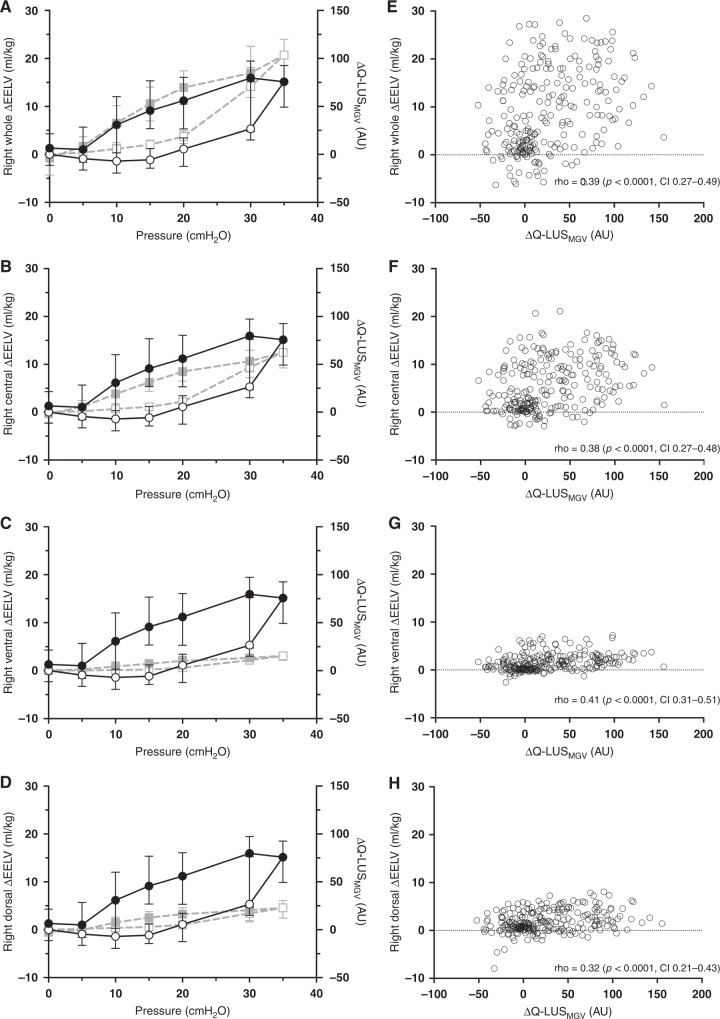
Dependent lung. **A**–**D** Static regional PV curves from EIT (squares, grey dashed line) of the whole right lung (**A**), ventral (**B**), central (**C**) and dorsal (**D**) regions and Q-LUS_MGV_ from dependent lung imaging (circles, black solid line). All data median (IQR). ∆Q-LUS_MGV_ normalised to baseline. Open shapes represent the inflation limb and closed shapes the deflation limb. **E**–**H** Correlation between ∆Q-LUS_MGV_ and regional lung volume for the corresponding lung regions in panels **A**–**D**. AU arbitrary units, CI confidence interval, cm H_2_O centimetres of water, EELV end-expiratory lung volume, rho Spearman’s correlation co-efficient, Q-LUS_MGV_ quantitative lung ultrasound mean grey value. Left axis: lung volume (ml/kg). Right axis: ∆Q-LUS_MGV_.

Q-LUS_MGV_ of the dependent lung correlated fairly with changes in regional volume (Fig. 4) of the right whole lung (rho = 0.39, *p* < 0.0001, CI 0.27–0.49), and central (rho = 0.38, *p* < 0.0001, CI 0.27–0.48), ventral (rho = 0.41, *p* < 0.0001, CI 0.31–0.51) and dorsal regions (rho = 0.32, *p* < 0.0001, CI 0.21–0.43).

#### Non-dependent LUS

**Fig. 5:**
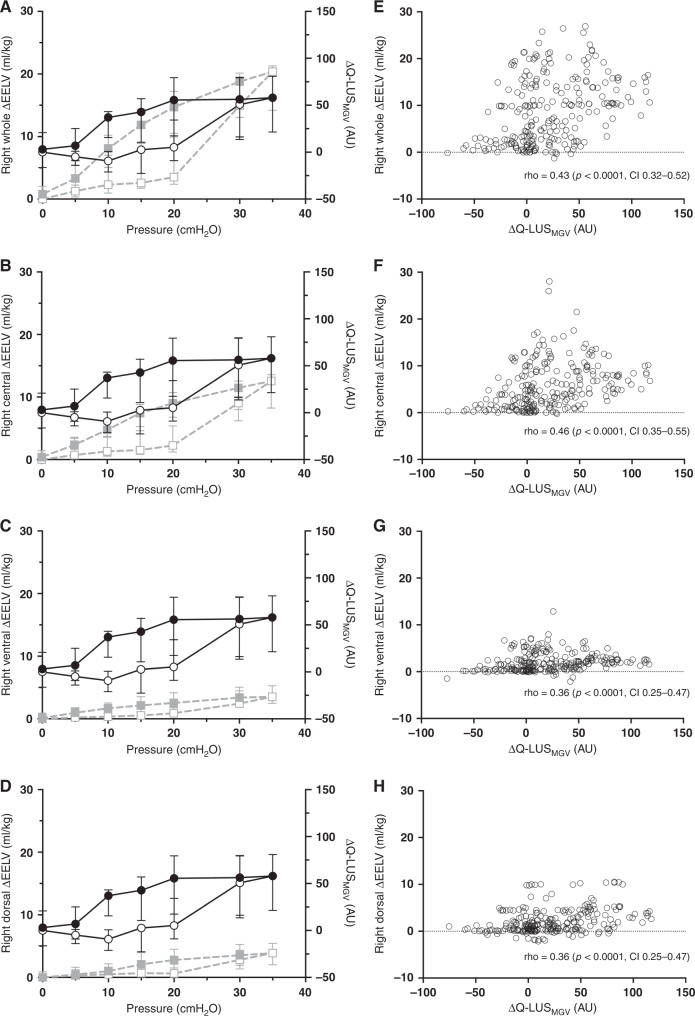
Non-dependent lung. **A**–**D** Static regional PV curves from EIT (squares, grey dashed line) of the whole right lung (**A**), ventral (**B**), central (**C**) and dorsal (**D**) regions and ∆Q-LUS_MGV_ from non-dependent lung imaging (circles, black solid line). All data median (IQR). ∆Q-LUS_MGV_ normalised to baseline. Open shapes represent the inflation limb and closed shapes the deflation limb. **E**–**H** Correlation between ∆Q-LUS_MGV_ and regional lung volume for the corresponding lung regions in panels **A**–**D**. AU arbitrary units, CI confidence interval, cm H_2_O centimetres of water, EELV end-expiratory lung volume, rho Spearman’s correlation co-efficient, Q-LUS_MGV_ quantitative lung ultrasound mean grey value. Left axis: lung volume (ml/kg). Right axis: ∆Q-LUS_MGV_.

Q-LUS_MGV_ of the non-dependent lung fairly correlated with changes in regional volume (Fig. 5) of the right whole lung (rho = 0.43, *p* < 0.001, CI 0.32–0.52), and central (rho = 0.46, *p* < 0.0001, CI 0.35–0.55), ventral (rho = 0.36, *p* < 0.0001, CI 0.25–0.47) and dorsal regions (rho = 0.36, *p* < 0.0001, CI 0.25–0.47).

### Interobserver agreement

There was excellent agreement between both investigators for the dependent (ICC = 0.87, 95% CI 0.83–0.90, Supplementary Fig. E5) and non-dependent lung Q-LUS_MGV_ (ICC = 0.93, 0.92–0.95, Supplementary Fig. E6).

### Modelling the pressure–volume relationships

The sigmoidal model of the PV relationship proposed by Venegas et al.^28^ was able to be fitted to the pooled inflation (adjusted *R*^2^ = 0.90, runs test; *p* = 0.80) and deflation series (adjusted *R*^2^ = 0.93, runs test; *p* = 0.54) derived from the Q-LUS_MGV_ of the dependent region (Supplementary Fig. E7), and from the pooled inflation (adjusted *R*^2^ = 0.98, runs test; *p* = 0.97) and deflation series (adjusted *R*^2^ = 0.92, runs test; *p* > 0.99) derived from the Q-LUS_MGV_ of the non-dependent region (Supplementary Fig. E8). Similarly, the model was able to be fitted to the pooled inflation (adjusted *R*^2^ = 0.99, runs test; *p* = 0.2) and deflation series (adjusted *R*^2^ = 0.99, runs test; *p* = 0.52) derived from EIT of the dependent region (Supplementary Fig. E9), and from the pooled inflation (adjusted *R*^2^ = 0.97, runs test; *p* = 0.54) and deflation series (adjusted *R*^2^ = 0.98, runs test; *p* = 0.33) derived from EIT of the non-dependent region (Supplementary Fig. E10). The predicted opening and closing pressures from both Q-LUS_MGV_ and EIT of the dependent lung (opening pressure; 25 cm H_2_O in both, closing pressure; 15 vs 12 cm H_2_O respectively, Supplementary Figs. E11 and E12) and the non-dependent lung (opening pressure; 25 cm H_2_O in both, closing pressure; 12 vs 15 cm H_2_O respectively, Supplementary Figs. E13 and E14) were similar.

## Discussion

As chest x-ray is a poor indicator of lung volume in preterm infants,^2^ neonatal clinicians are reliant on physiological approximations to guide lung recruitment.^30–32^ LUS may assist in guiding lung recruitment, but current LUS scoring systems poorly differentiate small changes in lung volume.^12^ In this experiment we evaluated the ability of mean greyscale analysis using computer-assisted quantitative lung ultrasound (Q-LUS_MGV_) to measure changes in lung volume. Q-LUS_MGV_ moderately correlated with changes in total and regional lung volume in preterm lambs and was able to detect the opening and closing pressures of the respiratory system, and pulmonary hysteresis in 38 of 40 animals. In addition, Q-LUS_MGV_ was able to detect relatively small changes in total lung volume during the deflation series. Finally, PV curves constructed from Q-LUS_MGV_ measurements demonstrated distinct inflation and deflation limbs that were able to be fitted to a known mathematical model of the PV relationship. Our findings suggest that Q-LUS_MGV_ detects key features of the PV relationship and may enhance the capability of LUS to monitor real-time changes in lung volume.

Quantitative ultrasound is an emerging field. Corradi et al. compared the accuracy of greyscale analysis to chest radiography, traditional LUS and CT diagnosis of pneumonia in adults.^33^ When compared to CT as a gold standard, quantitative LUS outperformed both chest radiography and traditional LUS in diagnosing pneumonia and significantly correlated with CT-detected non-aerated lung. The same authors demonstrated that when compared to pulmonary capillary wedge pressure and transpulmonary thermodilution, quantitative LUS outperformed traditional LUS in detecting pulmonary oedema in ventilated adults.^34^ Compared with previous studies comparing chest radiography to gold standard measurements of lung volume,^2^ Q-LUS_MGV_ exhibited a higher correlation with absolute measures of lung volume. This improved performance may be due to the dynamic nature of ultrasound imaging and the ability of Q-LUS_MGV_ to detect small changes in lung volumes that are not detected using other modalities. These findings suggest that Q-LUS_MGV_ supplementation may refine LUS detection of dynamic change lung volume, and subsequently guide lung recruitment.

Greyscale levels are influenced by ultrasound system configuration,^35^ which usually reflects individual operator preference. Consequently, configuration independent quantitative analysis measurements have been investigated.^13^ Using image textural feature analysis, Cobo et al. predicted neonatal respiratory morbidity from foetal LUS.^36–39^ Diagnostic accuracy was comparable to invasive testing requiring amniocentesis. Raimondi et al. analysed LUS images in preterm infants with respiratory distress and by combining both greyscale and textural features, the authors found a moderate correlation with oxygenation indices.^18^ Similarly, Tenorio et al. reported early prediction of white matter damage using textural analysis of cranial ultrasonography.^40^ In contrast, we opted for a simpler measurement, and to our knowledge, this is the first study to compare Q-LUS_MGV_ with dynamic changes in lung volume. Measurement of Q-LUS_MGV_ was performed with open-sourced image analysis software,^25^ negating the need for purpose-built programme development. Ultrasound settings were not altered between subjects, mitigating external influence from system settings. Standardisation of ultrasound settings and incorporating advanced analysis may improve the performance of this approach and warrants consideration in future studies.

The importance of placing ventilation on the deflation limb of the PV relationship is recognised.^26,41–44^ Using oxygenation as an approximation of lung volume, open lung ventilation strategies optimise lung volume by recruiting the lung to near total lung capacity using stepwise increases in continuous distending pressure, followed by gradual pressure reductions to place ventilation just above closing pressure of the lung.^30,42^ This process maps the quasistatic PV relationship of the respiratory system, delineating crucial points during the inflation and deflation series. An attractive alternative is to measure anatomical recruitment using bedside LUS. However, despite proven diagnostic utility,^5,8,9,45–48^ categorical LUS scoring systems^5,6^ are not designed to discriminate dynamic and small changes in lung volume.^12^ In contrast, Q-LUS_MGV_ is a continuous measurement, which in 8-bit depth can discriminate 255 increments of greyscale, exceeding human capabilities.^17^ Higher bit sampling may detect even smaller differences. In our study, Q-LUS_MGV_ identified key features of the PV relationship of the respiratory system in the preterm lung. Clear inflation and deflation limbs were able to be fitted to a known model of the PV relationship.^28^ Relatively small changes in lung volume were detected by significant changes in Q-LUS_MGV,_ particularly during the deflation series. Although this may be limited to homogenous lung disease, these findings support the hypothesis that objective measurements using image analysis may improve the ability of LUS to detect dynamic lung volume changes in real time.

Selective LUS imaging has shown promise in early homogenous lung disease;^6,9^ however, as gravity-dependent aeration distribution progresses, global imaging has increased diagnostic utility.^49,50^ As lung recruitment is a continuous process, selective imaging may be more practical. It was unknown whether selective Q-LUS_MGV_ correlates with changes in regional lung volume. We addressed this by comparing changes in regional lung volume derived from EIT to Q-LUS_MGV_. We demonstrated a fair correlation between Q-LUS_MGV_ and regional lung volumes in all areas of the lung. Despite smaller EELV being delivered to the ventral and dorsal lung, correlation was similar between regions. This was possibly due to relatively equal aeration secondary to the smaller absolute size of the dorsal and ventral regions and to homogenous lung pathology. In early respiratory distress syndrome secondary to surfactant deficiency, Q-LUS_MGV_ of a single region may reflect changes in both total and regional lung volume. Further studies are required to determine whether this is true in lung conditions associated with inhomogeneous aeration.

Our study has limitations. Application of our findings to human newborns remains to be established. However, the preterm lamb is a well-established model of the preterm lung with a strong track record of translation to human research.^21^ Imaging was limited to the right lung due to the rapid nature of PV relationship mapping and physical constraints. Whether whole lung Q-LUS_MGV_ more precisely detects changes in lung volume warrants further investigation. Greyscale image analysis currently is not available in real time, limiting the use of this method to offline measurements and research purposes. Development of this technique to function in real time is warranted. Another limitation of this study was that lambs were subject to a brief ventilation period followed by 30 min of apnoea on placental support, leading to significant collapse and potential lung fluid accumulation. It is possible that longer ventilation off placental support may lead to more lung recruitment and a wider range of Q-LUS_MGV_. Finally, although Q-LUS_MGV_ significantly correlated with lung volumes, inter-subject variability was notable. Incorporation of additional feature analysis may improve the precision of this technique.

Our study has strengths. This study is the largest LUS study using the preterm lamb as a model of the preterm lung  that compares LUS to gold standard, absolute and regional measurements of lung volume. The use of EIT is a novel addition, suggesting that in homogenous lung disease, changes in selected regional lung imaging are representative of changes in total lung volume. Finally, interobserver agreement between the two blinded investigators was excellent, suggesting that this measurement is reproducible between operators of varying levels of experience.

## Conclusions

Greyscale image analysis using computer-assisted quantitative LUS detected real-time changes in total and regional lung volume, and reliably mapped the PV relationship in the preterm lamb. This measurement may supplement traditional LUS in discerning dynamic changes in lung volume in preterm infants at the bedside. Further work is needed to improve the precision of this technique prior to clinical translation.

## Supplementary information

Author checklist

Supplementary information

## Data Availability

Individual animal data collected during the study and statistical analysis will be available beginning 3 months and ending 23 years after article publication to researchers who provide a methodologically sound proposal with approval by an independent review committee. Data will be available for analysis to achieve the aims in the approved proposal. Proposals should be directed to arun.sett@mcri.edu.au; to gain access, data requestors will need to sign a data access or material transfer agreement approved by the Murdoch Children’s Research Institute.
