# Supplementary material for: Quantitative lung ultrasound detects dynamic changes in lung recruitment in the preterm lamb
Source: Pediatr Res. 2022 Sep 27;93(6):1591–8. doi: 10.1038/s41390-022-02316-0 (PMC10172106; doi:10.1038/s41390-022-02316-0)
Supplement: Supplementary file 2 — Supplementary information [file 41390_2022_2316_MOESM2_ESM.pdf]

Quantitative lung ultrasound detects real time changes in total and regional lung volume in the preterm lamb – Online data supplement

Arun Sett[1,2,3,4,5], Gillian Foo[2], Kelly R Kenna[1], Rebecca J Sutton[1,8], Elizabeth J Perkins[1], Magdy Sourial[8], Sheryle R Rogerson[2,4,5], Brett J Manley[1,2,4], Peter G Davis[1,2,4], Prue M Pereira-Fantini[1,6], David G Tingay[1,2,6,7]

1. Neonatal Research, Murdoch Children's Research Institute, Victoria, Australia
2. Newborn Research Centre, The Royal Women's Hospital, Victoria
3. Joan Kirner Women's and Children's Hospital, Western Health, Victoria
4. Department of Obstetrics and Gynaecology, The University of Melbourne, Victoria
5. Paediatric Infant Perinatal Emergency Retrieval, The Royal Children's Hospital, Victoria
6. Department of Paediatrics, University of Melbourne, Victoria
7. Department of Neonatology, The Royal Children's Hospital, Victoria
8. Translational Research Unit, Murdoch Children's Research Institute, Victoria

Supplementary methods

This was a sub-study of a larger group of studies aiming to determine the impact of different pressure, flow and tidal volume strategies in the delivery room on lung injury. The study was approved by the Murdoch Children's Research Institute Animal Ethics Committee, Melbourne, Australia in accordance with National Health and Medical Research Council (Australia) guidelines and is reported as per the ARRIVE guidelines [1].

Animal preparation

Preterm lambs of 124–128 days gestation (term 140 days) were delivered from anaesthetized (isoflurane [0.5-1%], fentanyl [2-2.5 μg/kg/hr], ketamine [5 mg/kg/hr] and midazolam [1-1.5 mg/kg/hr]), betamethasone treated Border-Leicester/Merino ewes via cesarian section. Lambs were exteriorized and a saline filled glove was placed over the fetal head to prevent lung liquid loss. A carotid artery and a jugular vein catheter were inserted to monitor blood gas status and administer fluids as required. A custom built 32-electrode electrical impedance tomography (EIT) belt (Swisstom AG, Landquart, Switzerland) [2,3] was fitted around the chest. After intubation with a 4.0 mm cuffed endotracheal tube and passive lung liquid drainage, ventilation was commenced on placental support using the randomly assigned ventilation protocol as part of the primary studies. Lambs were ventilated with using pressure-controlled, time-cycled ventilation using a SLE5000 ventilator (SLE UK Ltd, South Croydon, UK). Ventilation parameters consisted of positive end expiratory pressure (PEEP); 8 centimeters of water (cm H_2O), maximal peak inspiratory pressure (P_{max}); 30 - 50 cm H_2O titrated to achieved tidal volume and lung compliance, targeted tidal volume (TTV); 3 – 7 ml/kg, rate; 60 breaths per minute, inspiratory time (Ti); 0.45 seconds, FiO_2; 0.21, bias flow; 4 – 8 L/min. All lambs received a dynamic PEEP open lung manoeuver prior to initiation of ventilation [2]. After 15 minutes ventilation was ceased, the endotracheal tube was clamped and lambs were maintained

on placental support for 30 minutes whilst apneic to allow sufficient time for upregulation of early markers of lung injury [4,5]. At conclusion, the lung was opened to atmosphere for 2-4 minutes and the static PV relationship of the respiratory system was mapped using a 200 mL calibrated glass syringe with pre-defined pressure increments from atmosphere (0 cm H_2O) to maximal inflation pressure (35 cm H_2O) [2]. The lung was held at each pressure step until volume stability was achieved or 30 seconds, whichever was first. Opening and closing pressure was identified as the steepest lower inflection point on the inflation limb and steepest upper inflection point on the deflation limb respectively. On completion, the umbilical cord was clamped and a lethal dose of sodium pentobarbitone (100 mg/kg) was administered.

Lung ultrasound (LUS)

LUS was performed simultaneously with mapping of the PV relationship of the respiratory system using a Logiq E (GE Healthcare, Wauwatosa, WI, USA) and Terason USMART 3200T (Terason, Burlington, MA, USA) ultrasound systems with a 12-megahertz broadband high-frequency linear transducer. Depth was set to 2.5 cm and the focal zone positioned at the pleural line. Gain was set to 40 decibels and not adjusted between animals. Filters were deactivated. Dependent and non-dependent lung images were acquired from the right lower lateral and right anterior thorax respectively. The transducer was orientated longitudinally to capture 3 rib spaces. The transducer was adjusted to be perpendicular to the skin surface as indicated by maximal pleural line intensity and rib shadowing. Three second videos at each pressure increment of 0, 5, 10, 15, 20, 30 and 35 cm H_2O during the inflation and deflation series were acquired. All LUS images were stored under a unique study number with no details of the measured pressures or volumes.

Image analysis

Single channel, uncompressed ultrasound images from each pressure increment were de-identified and imported into FIJI, ImageJ (National Institute of Health, Bethesda, Maryland, USA) in Digital Imaging and Communications in Medicine (DICOM) format [6]. Cine loops were screened and representative still images were selected and converted to 8-bit format for measurement of the Q-LUS_{MGV} of the pleural region of interest (ROI). Two investigators (A.S., 4 years LUS experience and G.F., 1 year LUS experience) manually delineated the pleural ROI on de-identified, randomized images. The depth of the pleural region was defined based on previous reports of normal pleural line thickness in preterm infants [7]. The inferior border of the pleural ROI was drawn at a 50 pixel depth perpendicular to the superior margin. Q-LUS_{MGV} was determined by measuring the sum grey value of all the pixels within the ROI and dividing the result by the total number of pixels within the ROI [6] using the built-in measurement package. An 8-bit pixel depth permitted a Q-LUS_{MGV} range from 0 (black) to 255 (white). To construct pressure volume (PV) curves from Q-LUS_{MGV}, measurements were normalized to the baseline measurement at 0 cm H_2O and the relative change in Q-LUS_{MGV} was plotted per pressure increment. Q-LUS_{MGV} were normalized to baseline as although the ETT is left open to air, residual air will remain in the lung and this will vary between subjects depending on lung mechanics. Individual PV curves were constructed from raw values and pooled curves from each group were constructed using the median (IQR) measurements.

Electrical impedance tomography

EIT images were continuously sampled at 48 frames/second throughout mapping of the static PV relationship of the respiratory system. Data were reconstructed using an anatomically

correct finite element model of the lamb thorax filtered to the respiratory domain [8]. The time-course EIT signal for the whole lung was calibrated against the volume changes measured by the super syringe [2], and the lung volumes of the right whole lung, dorsal, central and ventral regions were determined from weighting the pixel distribution of each region to the calibrated whole lung volumes [9,10]. The lung regions were delineated by 3 equally sized portions (33% of the total individual lung volume). Hence, each lung contained a variable amount of lung tissue due to the shape of the chest.

		Pressure ($cm\ H_2O$)											
		Inflation Series							Deflation Series				
		0	5	10	15	20	30	35	30	20	15	10	5
Pressure ($cm\ H_2O$) Inflation Series	5	ns											
	10	ns	ns										
	15	ns	ns	ns									
	20	ns	ns	ns	ns								
	30	0.02	0.04	0.01	0.01	ns							
	35	0.01	0.01	0.01	0.01	0.01	ns						
Deflation Series	30	0.02	0.02	0.02	0.02	0.03	ns	ns					
	20	0.01	0.01	0.01	0.01	0.01	ns	ns	ns				
	15	0.01	0.01	0.01	0.01	0.01	ns	ns	ns	ns			
	10	ns	ns	ns	ns	ns	ns	ns	ns	0.04	0.06		
	5	ns	ns	ns	ns	ns	ns	ns	ns	ns	ns	ns	
	0	ns	ns	ns	ns	ns	ns	<0.01	0.03	0.01	0.01	0.03	ns

Supplementary Table E1: Results for the post-hoc Wilcoxon signed rank some test with a Bonferroni correction for multiple comparisons of Q-LUS_{MGV} from dependent imaging. Significant results are highlighted in blue. Results approaching significant are highlighted in orange. ns; not significant. Green dashed line; opening pressure, red dashed line; closing pressure.

		Pressure ($cm\ H_2O$)											
		Inflation Series							Deflation Series				
		0	5	10	15	20	30	35	30	20	15	10	5
Pressure ($cm\ H_2O$) — Inflation Series	5	ns											
	10	ns	ns										
	15	ns	ns	ns									
	20	ns	ns	ns	ns								
	30	0.01	0.02	0.01	0.02	0.01							
	35	<0.01	<0.01	<0.01	0.01	0.01	ns						
Deflation Series	30	0.01	<0.01	0.01	0.03	0.04	ns	ns					
	20	0.01	0.01	0.02	0.03	0.04	ns	ns	ns				
	15	0.01	0.01	0.01	0.02	0.05	ns	ns	ns	ns			
	10	<0.01	<0.01	<0.01	0.01	0.05	ns	ns	ns	ns	ns		
	5	ns	ns	ns	ns	ns	0.02	0.01	0.03	0.01	0.02	0.01	
	0	ns	ns	ns	ns	ns	0.02	<0.01	0.02	0.01	<0.01	<0.01	ns

Supplementary Table E2: Results for the post-hoc Wilcoxon signed rank some test with a Bonferroni correction for multiple comparisons of Q-LUS$_{MGV}$ non-dependent lung imaging. Significant results are highlighted in blue. Results approaching significance are highlighted in orange. ns; not significant. Green dashed line; opening pressure, red dashed line; closing pressure.

Supplementary figures

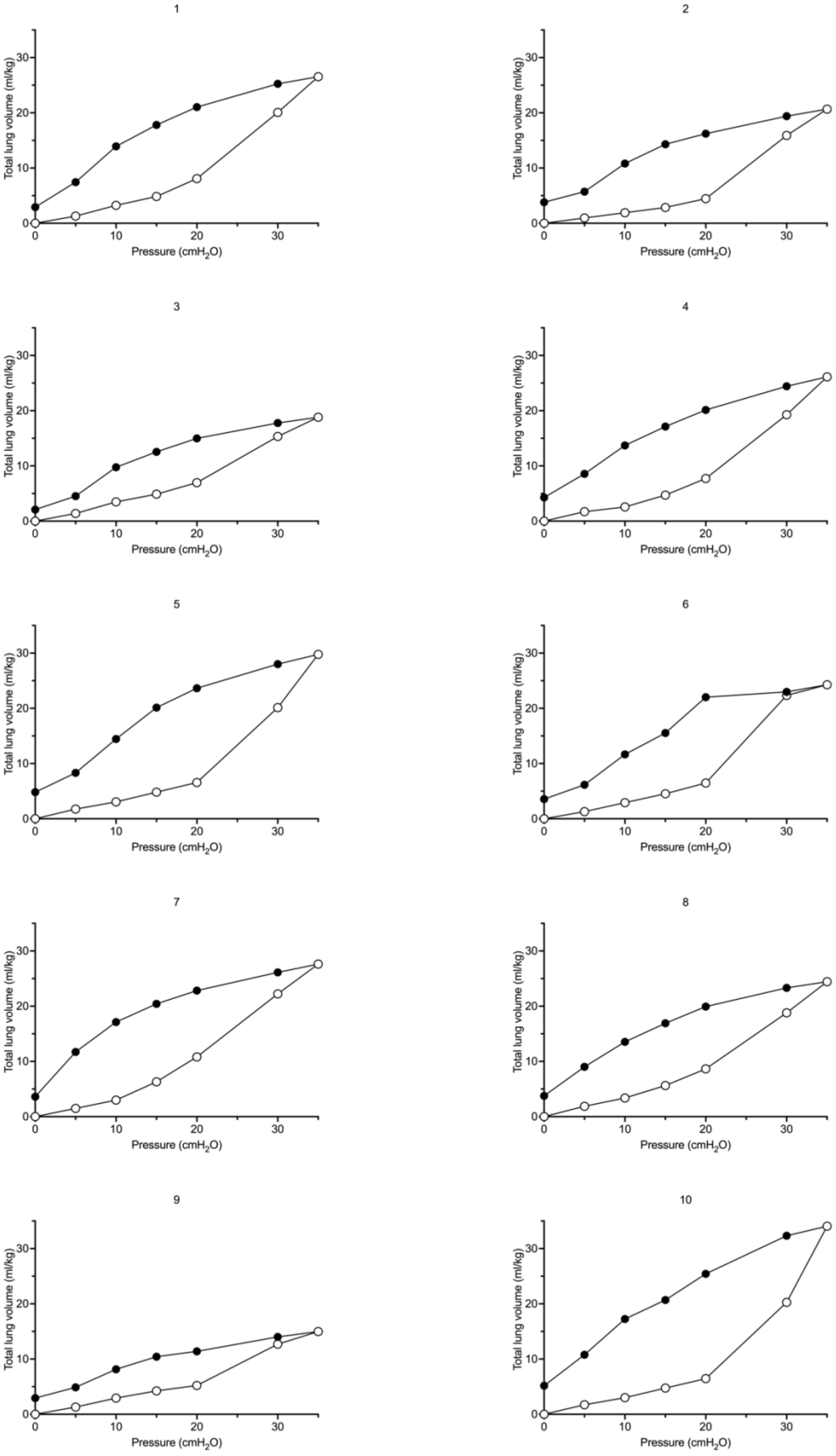

Supplementary Figure E1A:

Individual PV curves for dependent imaging lambs 1 to 10. Hysteresis is seen in all lambs. Opening and closing pressure occurs at 20 and 10 cm H_2O respectively in all lambs except lamb 6. Open circles; inflation series. Closed circles; deflation series.

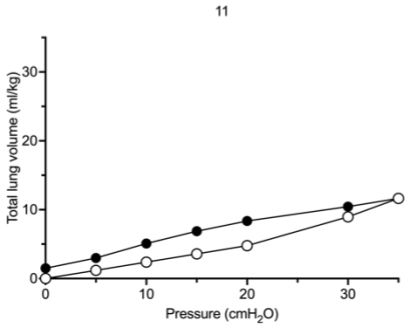
11
Total lung volume (ml/kg)
0
10
20
30
Pressure (cmH_2O)

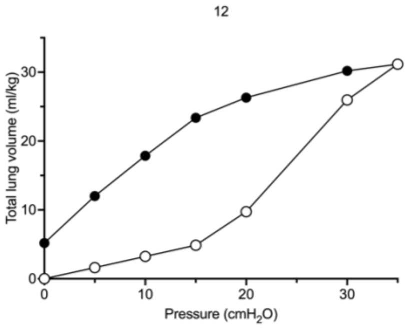
12
Total lung volume (ml/kg)
0
10
20
30
Pressure (cmH_2O)

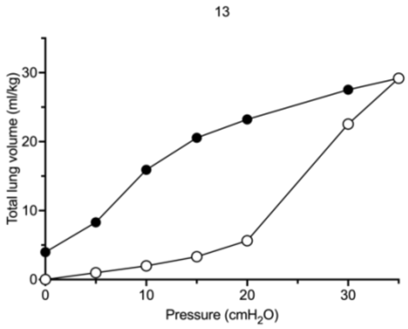
13
Total lung volume (ml/kg)
0
10
20
30
Pressure (cmH_2O)

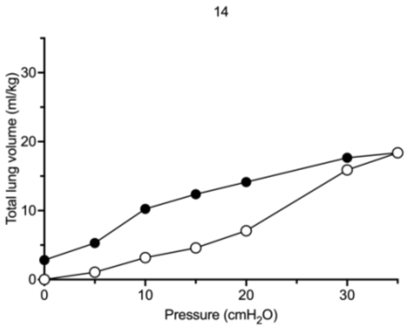
14
Total lung volume (ml/kg)
0
10
20
30
Pressure (cmH_2O)

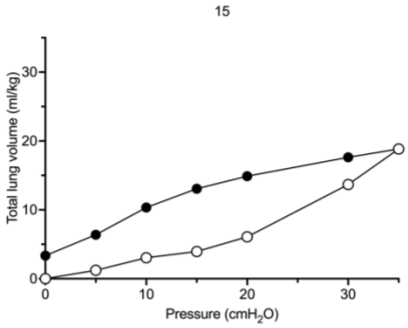
15
Total lung volume (ml/kg)
0
10
20
30
Pressure (cmH_2O)

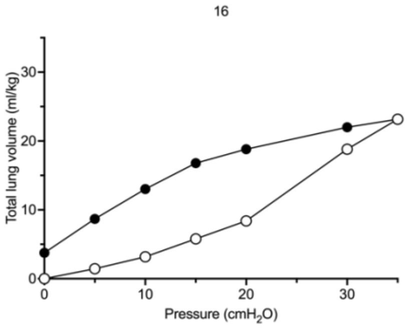
16
Total lung volume (ml/kg)
0
10
20
30
Pressure (cmH_2O)

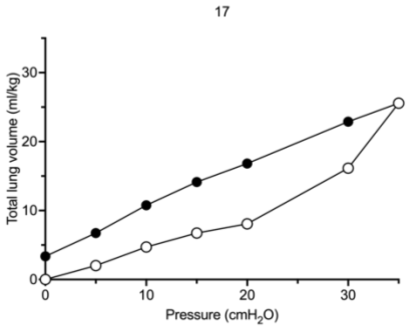
17
Total lung volume (ml/kg)
0
10
20
30
Pressure (cmH_2O)

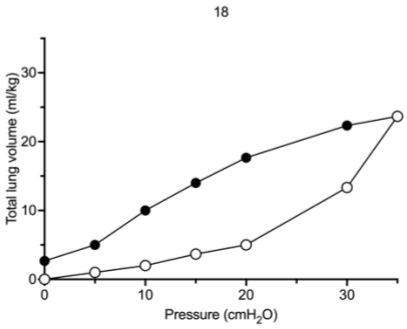
18
Total lung volume (ml/kg)
0
10
20
30
Pressure (cmH_2O)

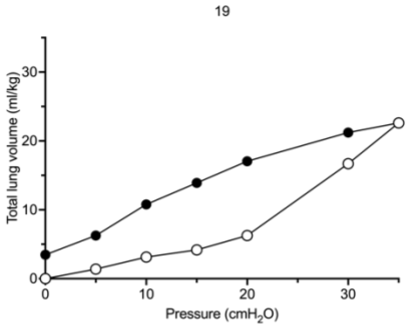
19
Total lung volume (ml/kg)
0
10
20
30
Pressure (cmH_2O)

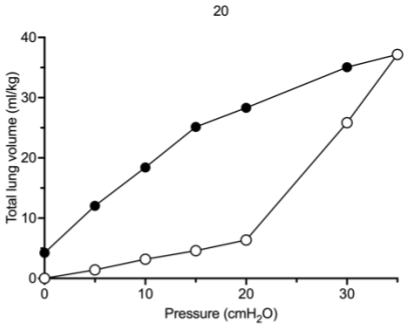
20
Total lung volume (ml/kg)
0
10
20
30
40
Pressure (cmH_2O)

Supplementary Figure E1B:

Individual PV curves for dependent imaging lambs 11 to 20. Hysteresis is seen in all lambs. Opening and closing pressure occurs at 20 and 10 cm H_2O respectively in all lambs. Open circles; inflation series. Closed circles; deflation series

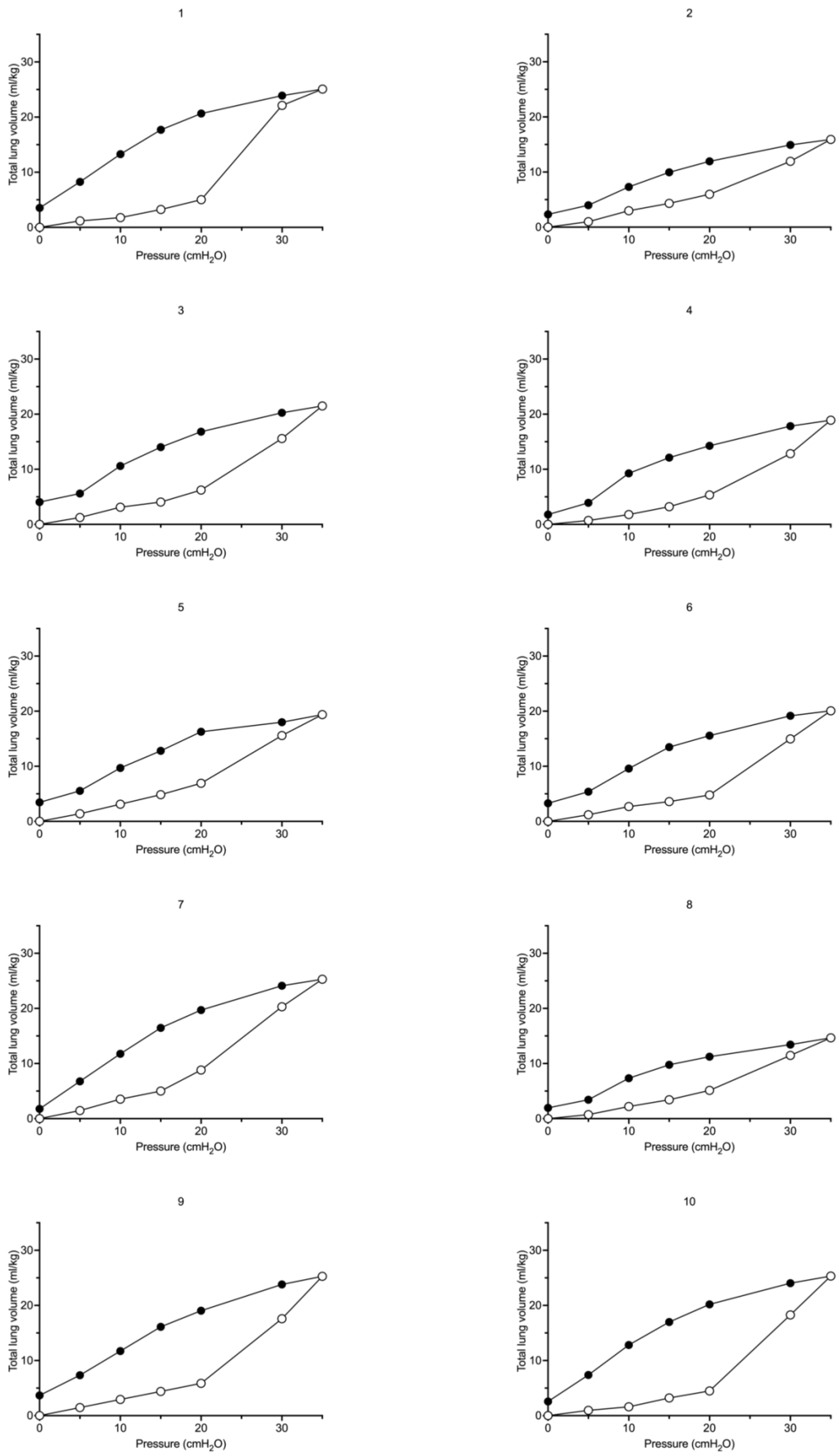
1
2
3
4
5
6
7
8
9
10
Total lung volume (ml/kg)
Pressure (cmH2O)
0
10
20
30

Supplementary Figure E2A:

Individual PV curves for non-dependent imaging lambs 1 to 10. Hysteresis is seen in all lambs. Opening at 20 cm H_2O in all lambs. Closing pressure occurs at 10 cm H_2O in all lambs except lamb 5. Open circles; inflation series. Closed circles; deflation series.

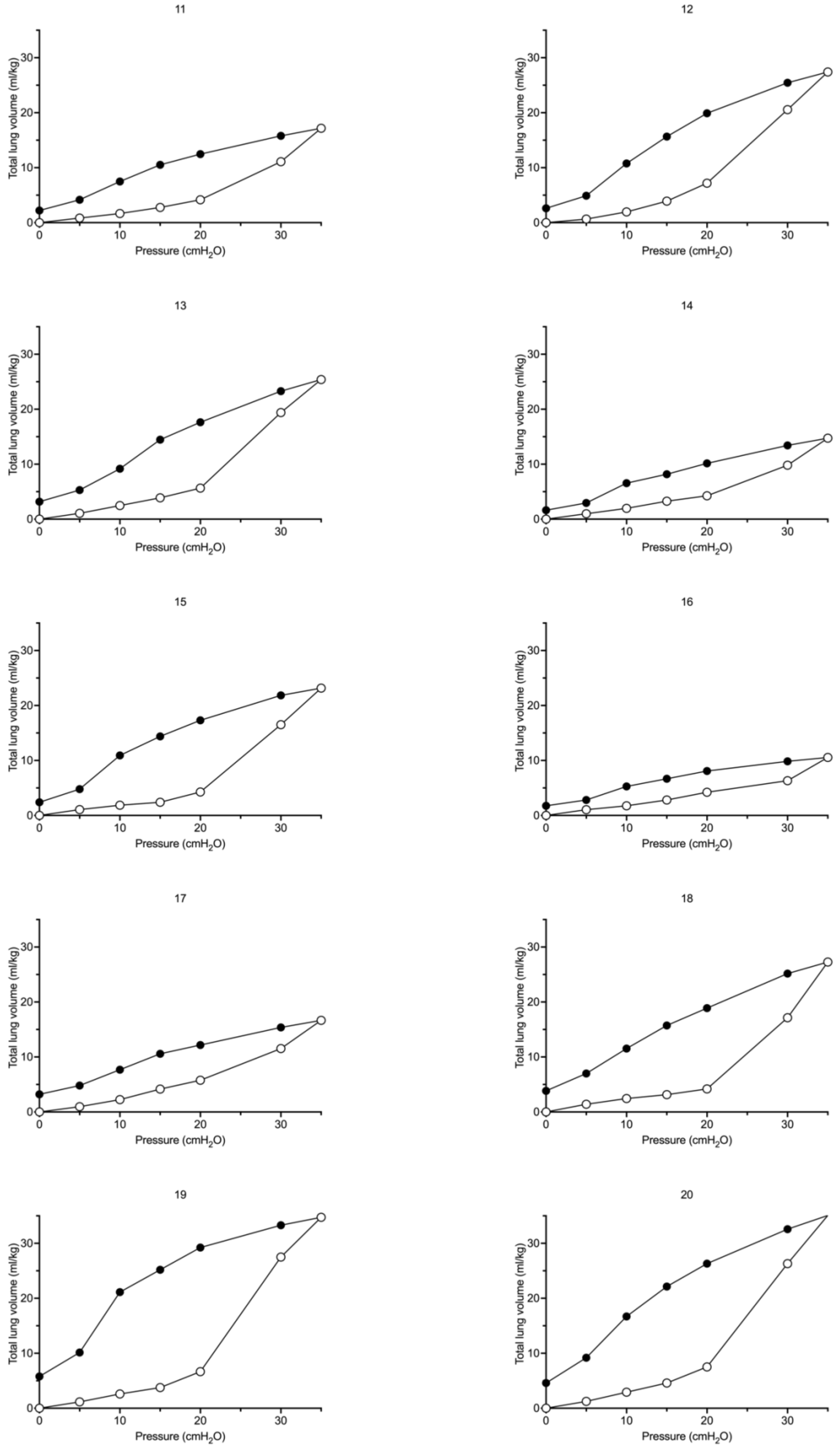
11
12
13
14
15
16
17
18
19
20
Total lung volume (ml/kg)
Pressure (cmH$_2$O)

Supplementary Figure E2B:

Individual PV curves for non-dependent imaging lambs 11 to 20. Hysteresis is seen in all lambs. Opening at 20 cm H_2O in all lambs except lamb 16. Closing pressure occurs at 10 cm H_2O in lambs except lamb 13. Open circles; inflation series. Closed circles; deflation series.

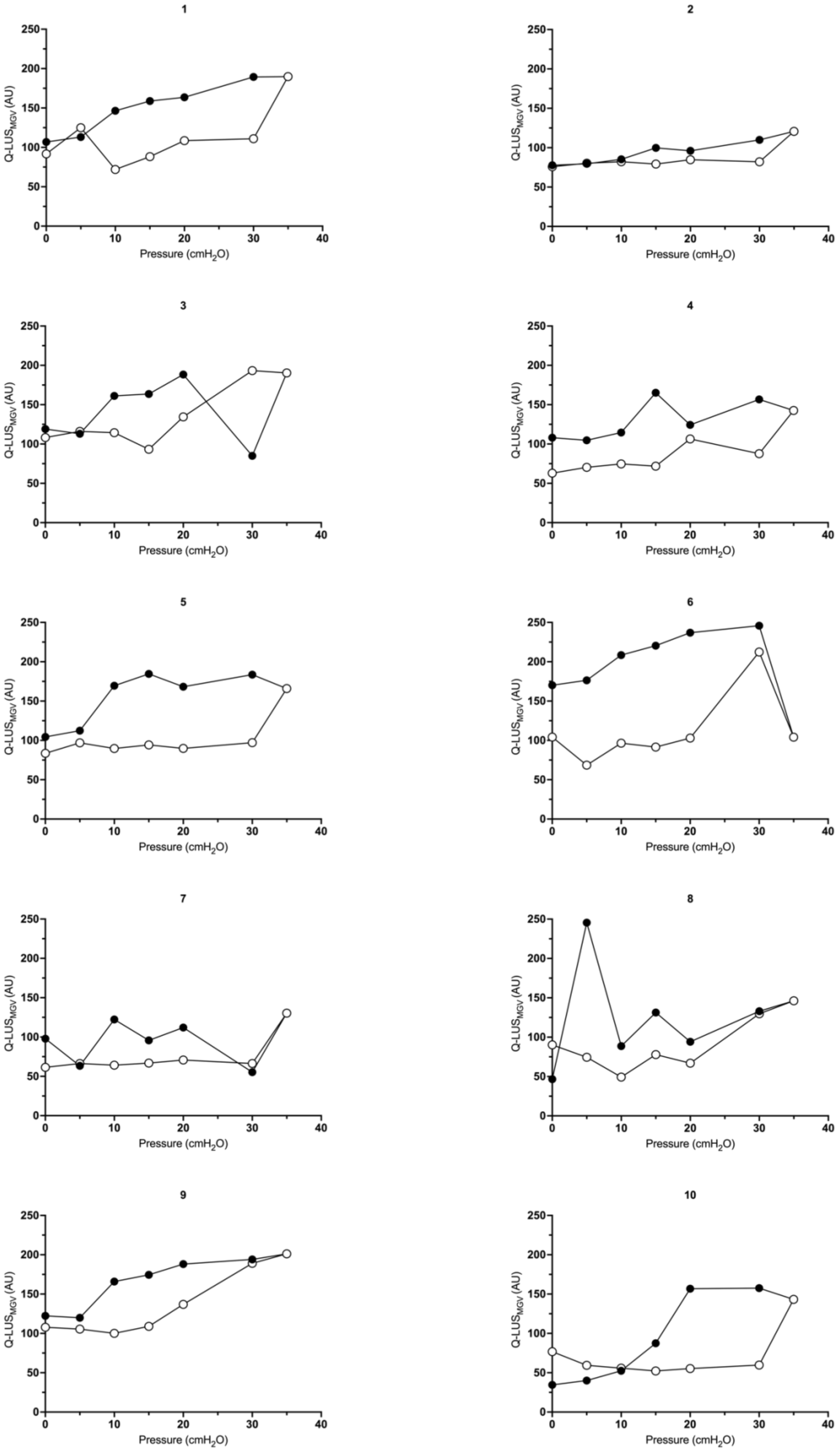

1
2
3
4
5
6
7
8
9
10
Q-LUS$_{MGV}$ (AU)
Pressure (cmH$_2$O)
0
50
100
150
200
250
0
10
20
30
40

Supplementary Figure E3A:

Individual pressure / Q-LUS$_{MGV}$ curves from dependent imaging lambs 1-10. Distinct inflation and deflation limbs, and hysteresis is demonstrated in all lambs. Open circles; inflation series. Closed circles; deflation series. AU; Arbitrary units, Q-LUS$_{MGV}$; Quantitative lung ultrasound mean grey value.

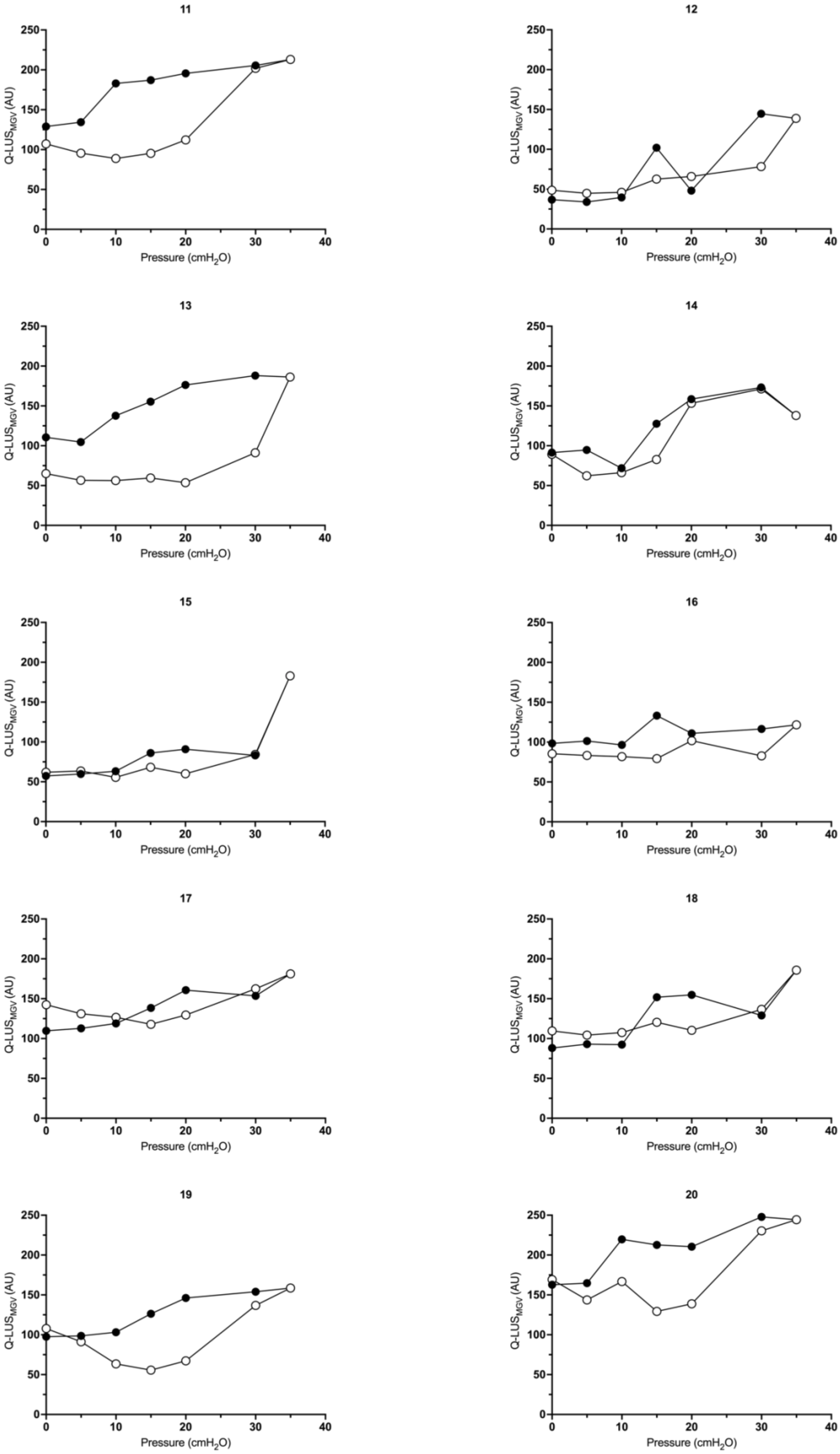
11
12
13
14
15
16
17
18
19
20
Q-LUS$_{MGV}$ (AU)
Pressure (cmH$_2$O)
0
50
100
150
200
250
0
10
20
30
40

Supplementary Figure E3B:

Individual pressure / Q-LUS_{MGV} curves from dependent imaging lambs 11-20. Distinct inflation and deflation limbs, and hysteresis is demonstrated in all lambs. Open circles; inflation series. Closed circles; deflation series. Q-LUS_{MGV}; AU; Arbitrary units, Quantitative lung ultrasound mean grey value.

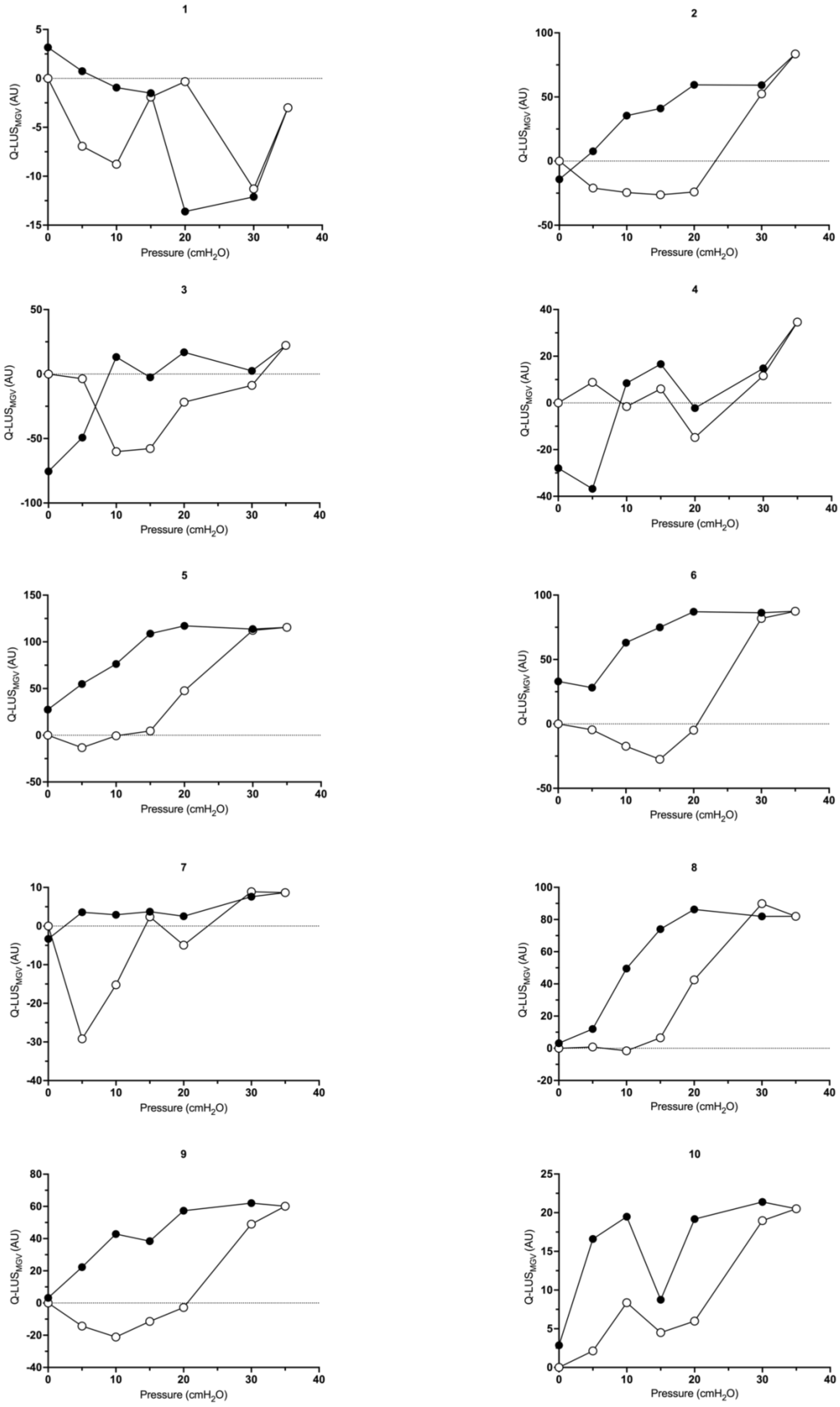
1
2
3
4
5
6
7
8
9
10
Q-LUS$_{MGV}$ (AU)
Pressure (cmH$_2$O)

Supplementary Figure E4A:

Individual pressure / Q-LUS$_{MGV}$ relationships from non-dependent imaging lambs 1-10. Distinct inflation and deflation limbs, and hysteresis is demonstrated in all lambs except lamb 1. Open circles; inflation series. Closed circles; deflation series. AU; Arbitrary units, Q-LUS$_{MGV}$; Quantitative lung ultrasound mean grey value.

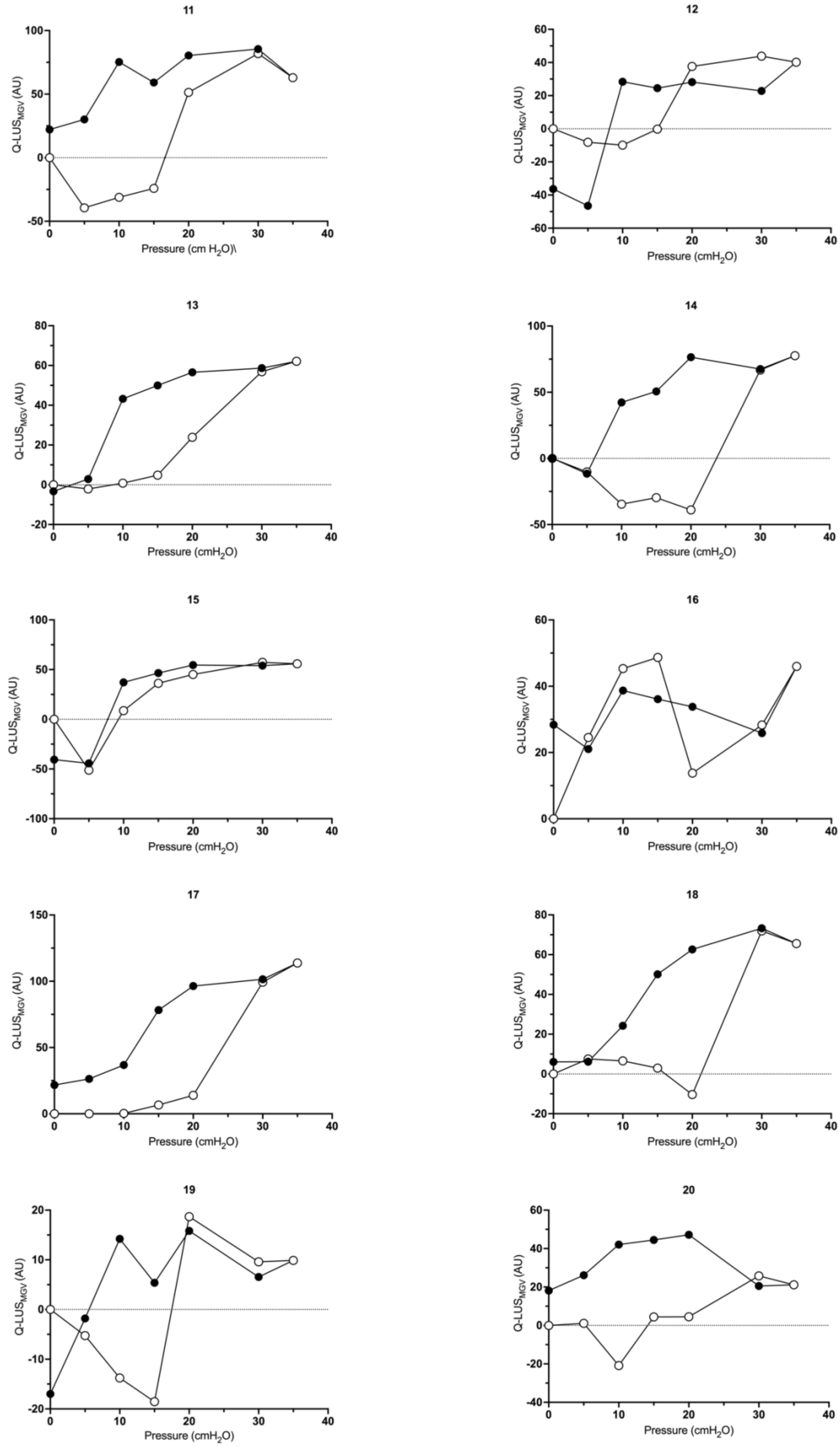
11
12
13
14
15
16
17
18
19
20
Q-LUS$_{MGV}$ (AU)
Pressure (cm H$_2$O)\
Pressure (cmH$_2$O)

Supplementary Figure E4B:

Individual pressure / Q-LUS_{MGV} relationships from non-dependent imaging lambs 1-10. Distinct inflation and deflation limbs, and hysteresis is demonstrated in all lambs except lamb 16. Open circles; inflation series. Closed circles; deflation series. AU; Arbitrary units, Q-LUS_{MGV}; Quantitative lung ultrasound mean grey value.

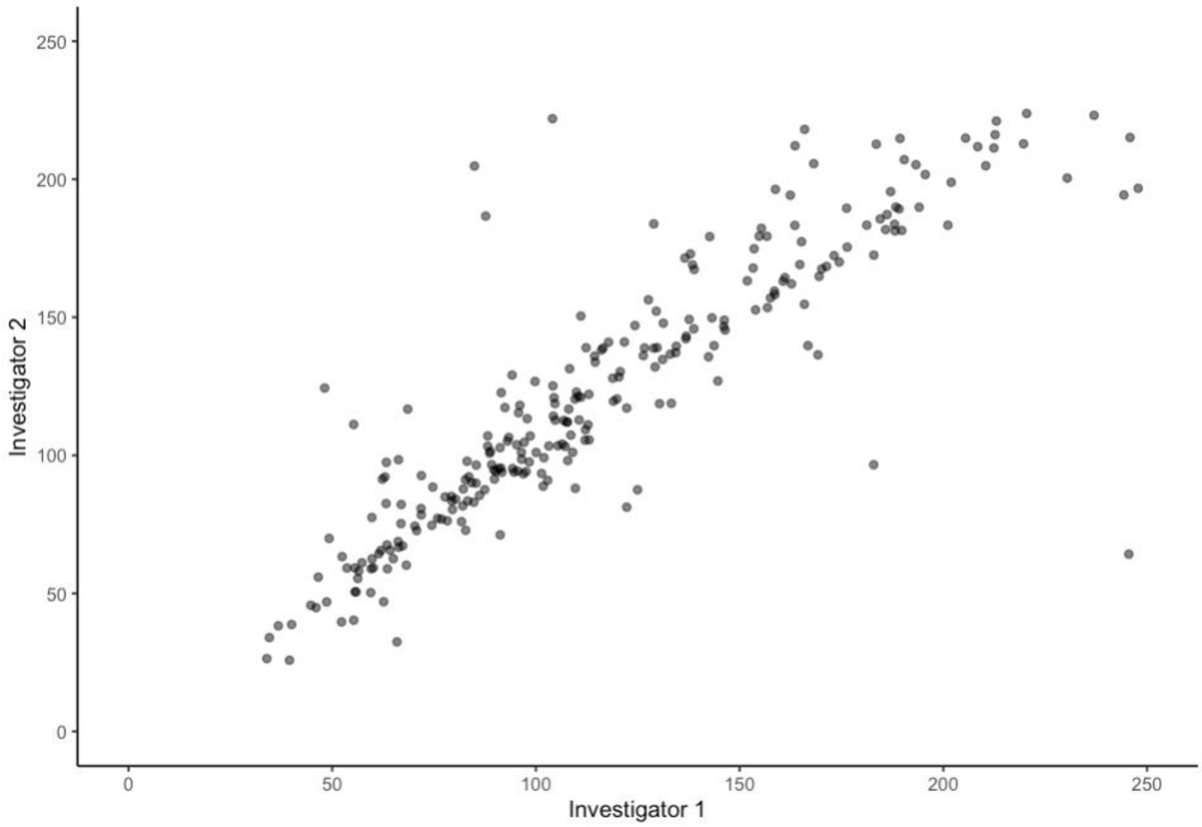

Supplementary Figure E5: Q-LUS$_{MGV}$ measurements of the dependent lung from two investigators who were unaware of the pressures and volumes. X and Y axis: Q-LUS$_{MGV}$ (AU). Grey dots represent individual measurements.

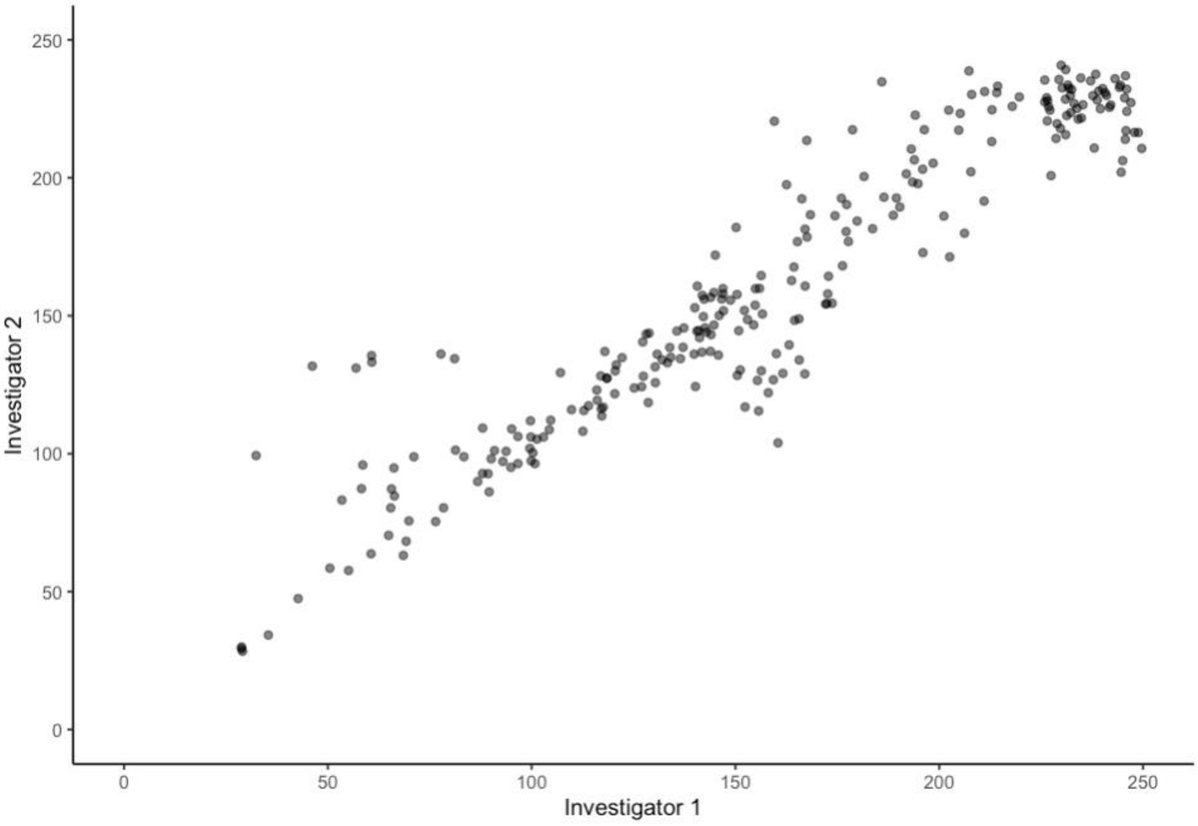

Supplementary Figure E6: Q-LUS$_{MGV}$ measurements of the non-dependent lung from two investigators who were unaware of the pressures and volumes. X and Y axis: Q-LUS$_{MGV}$ (AU). Grey dots represent individual measurements.

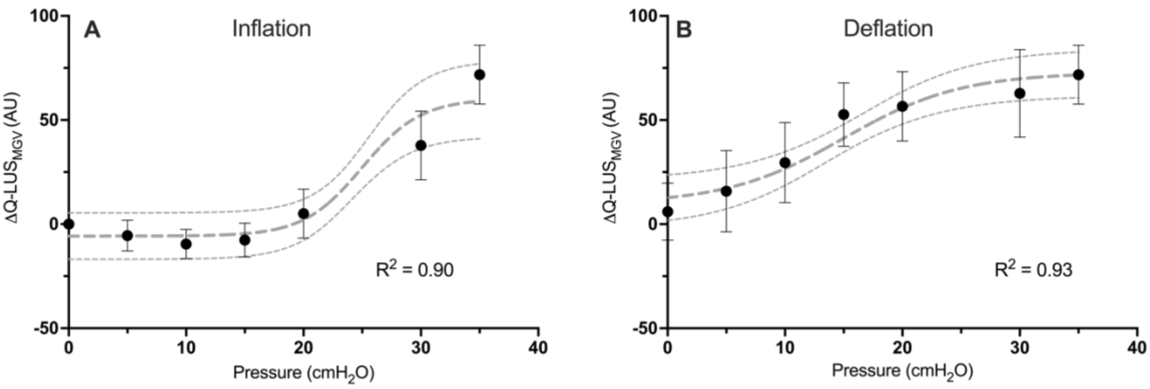

Supplementary Figure E7: Right dependent lung. Pressure/ΔQ-LUS$_{MGV}$ relationship of the inflation (A) and deflation (B) series using data from 20 lambs where the right dependent lung was imaged. Large grey dashed line; The inflation and deflation data pairs fitted to the sigmoidal model proposed by Venegas and colleagues. Small grey dashed line; 95% CI bands for model. All data represented as Mean (95% CI error bars). AU; Arbitrary units, CI; confidence interval, cm H2O; centimetres of water, Q-LUS$_{MGV}$; Quantitative lung ultrasound mean grey value.

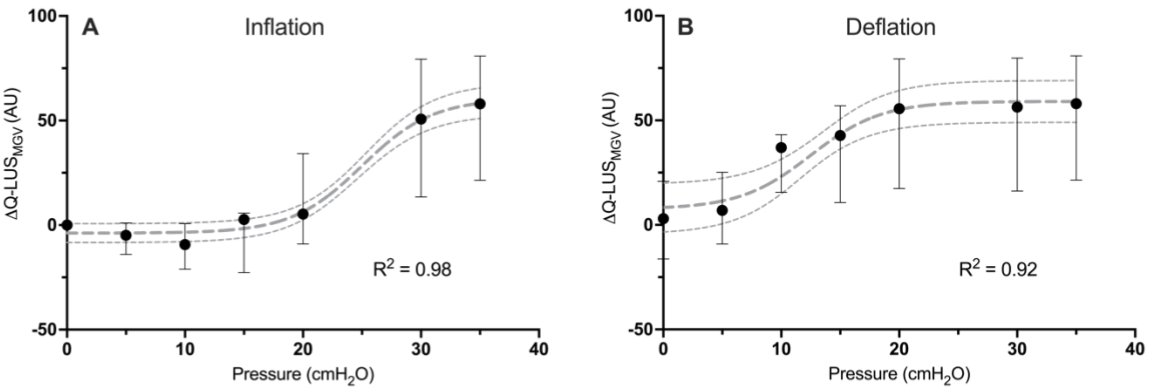

Supplementary Figure E8: Right non-dependent lung. Pressure/ΔQ-LUS_{MGV} relationship of the inflation (A) and deflation (B) series using data from 20 lambs where the right non-dependent lung was imaged. Large grey dashed line; The inflation and deflation data pairs fitted to the sigmoidal model proposed by Venegas and colleagues. Small grey dashed line; 95% CI bands for model. All data represented as Mean (95% CI error bars). AU; Arbitrary units, CI; confidence interval, cm H2O; centimetres of water, Q-LUS_{MGV}; Quantitative lung ultrasound mean grey value.

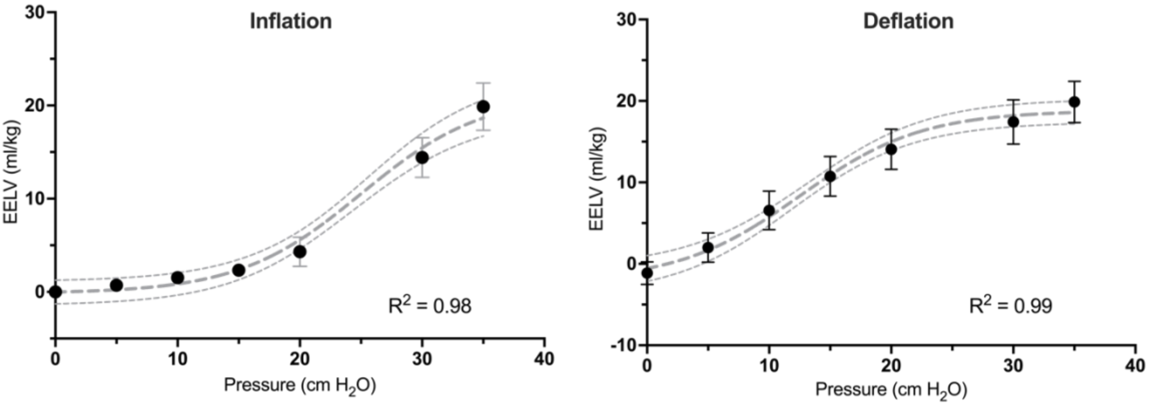

Supplementary Figure E9: Whole right lung pressure/EELV relationship of the inflation (A) and deflation (B) series using data from 20 lambs in the dependent imaging group. Large grey dashed line; The inflation and deflation data pairs fitted to the sigmoidal model proposed by Venegas and colleagues. Small grey dashed line; 95% CI bands for model. All data represented as Mean (95% CI error bars). EELV; end expiratory lung volume.

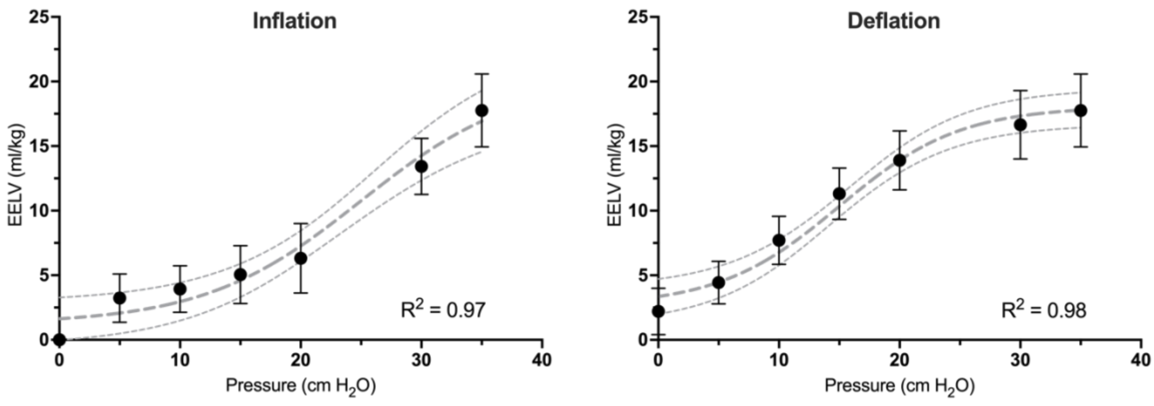

Supplementary Figure E10: Whole right lung pressure/EELV relationship of the inflation (A) and deflation (B) series using data from 20 lambs in the non-dependent imaging group. Large grey dashed line; The inflation and deflation data pairs fitted to the sigmoidal model proposed by Venegas and colleagues. Small grey dashed line; 95% CI bands for model. All data represented as Mean (95% CI error bars). EELV; end expiratory lung volume.

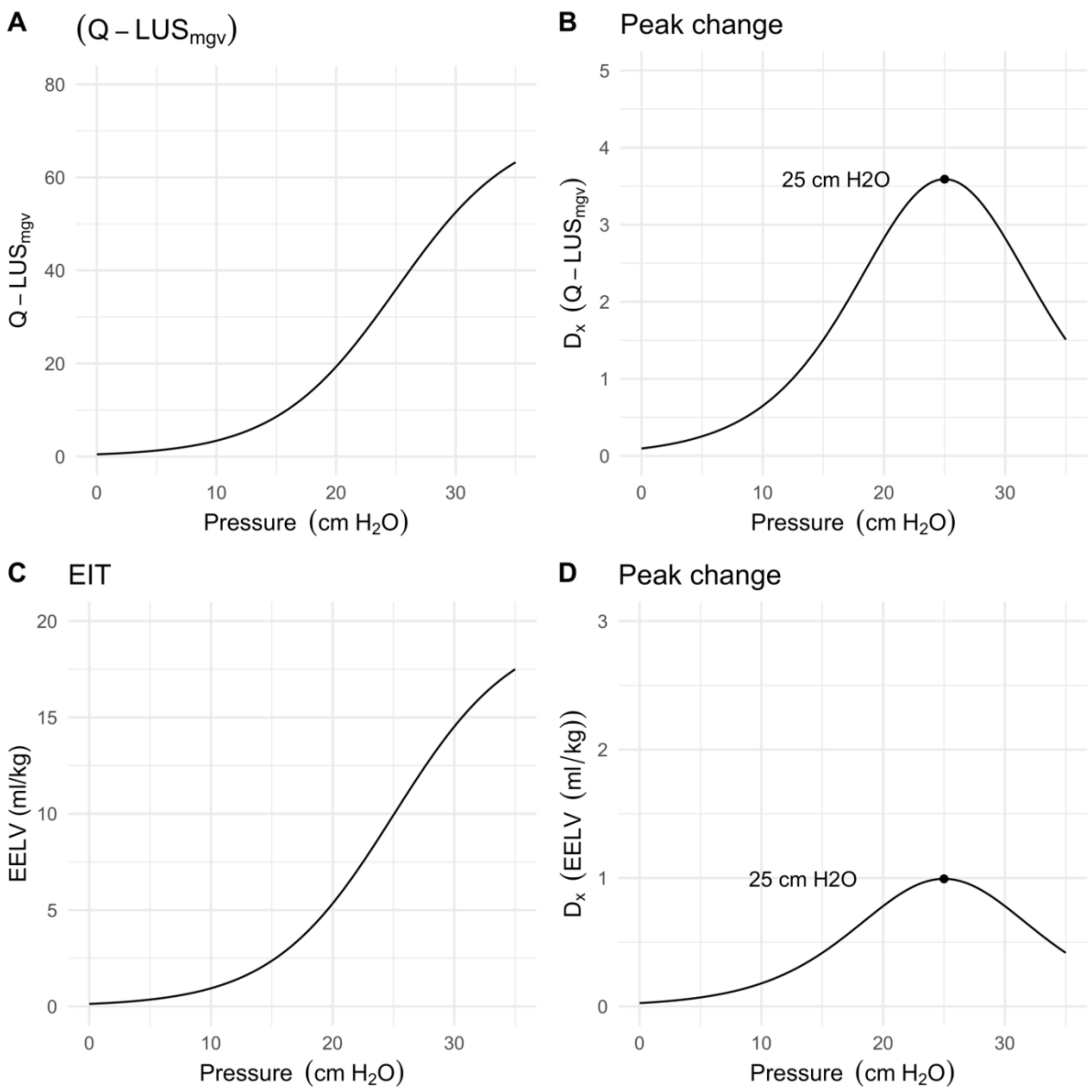

Supplementary Figure E11: Predicted opening pressure from fitting the mathematical model proposed by Venegas et al to inflation curves constructed from dependent Q-LUS$_{MGV}$ (A) and right whole lung EIT (C) respectively. Differential curves demonstrating peak change (black dot) in Q-LUS$_{MGV}$ (C) and EELV (D) are shown on the right. Opening pressure is at 25 cm H_2O in both. EELV; End expiratory lung volume, Q-LUS$_{MGV}$; Quantitative lung ultrasound mean grey value. Pressure rounded to nearest whole number. Y axis scale adjusted per figure for ease of visualization.

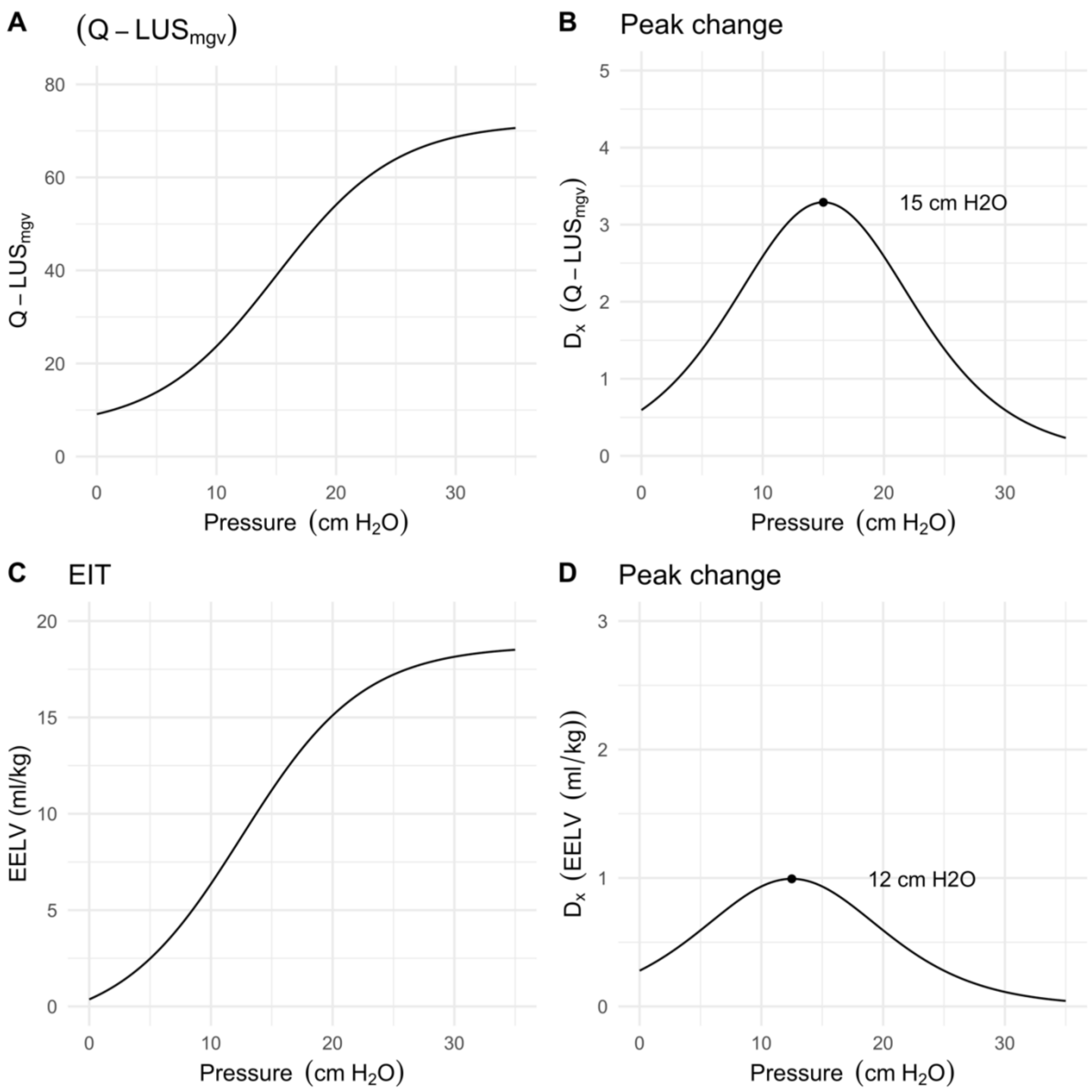

Supplementary Figure E12: Predicted closing pressure from fitting the mathematical model proposed by Venegas et al to deflation curves constructed from dependent Q-LUS$_{MGV}$ (A) and right whole lung EIT (C) respectively. Differential curves demonstrating peak change (black dot) in Q-LUS$_{MGV}$ (C) and EELV (D) are shown on the right. Closing pressure is at 15 cm H_2O and 12 cm H_2O from Q-LUS$_{MGV}$ and EIT respectively. EELV; End expiratory lung volume, Q-LUS$_{MGV}$; Quantitative lung ultrasound mean grey value. Pressure rounded to nearest whole number. Y axis scale adjusted per figure for ease of visualization.

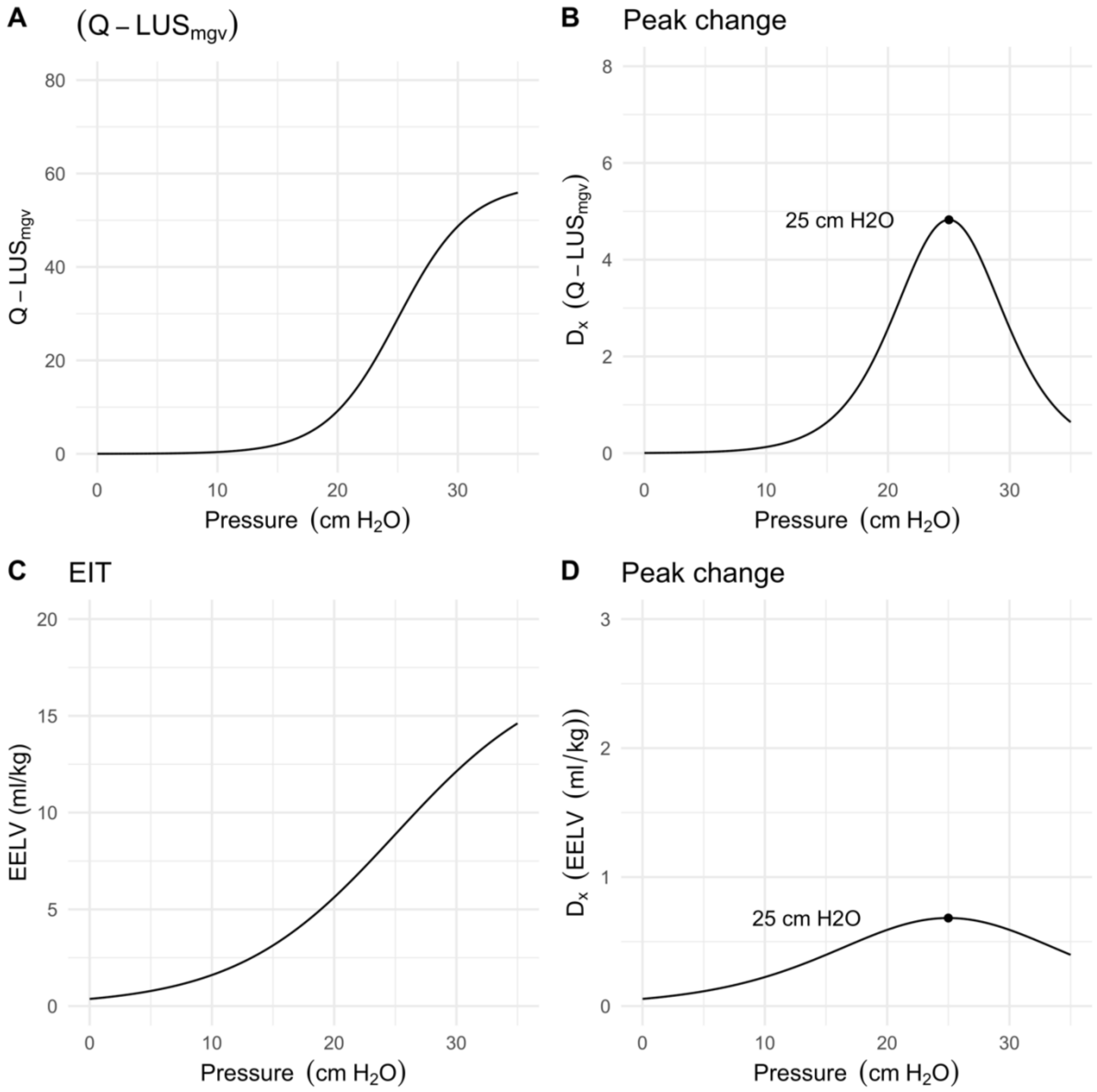

Supplementary Figure E13: Predicted opening pressure from fitting the mathematical model proposed by Venegas et al to inflation curves constructed from non-dependent Q-LUS$_{MGV}$ (A) and right whole lung EIT (C) respectively. Differential curves demonstrating peak change (black dot) in Q-LUS$_{MGV}$ (C) and EELV (D) are shown on the right. Opening pressure is at 25 cm H_2O in both. EELV; End expiratory lung volume, Q-LUS$_{MGV}$; Quantitative lung ultrasound mean grey value. Pressure rounded to nearest whole number. Y axis scale adjusted per figure for ease of visualization.

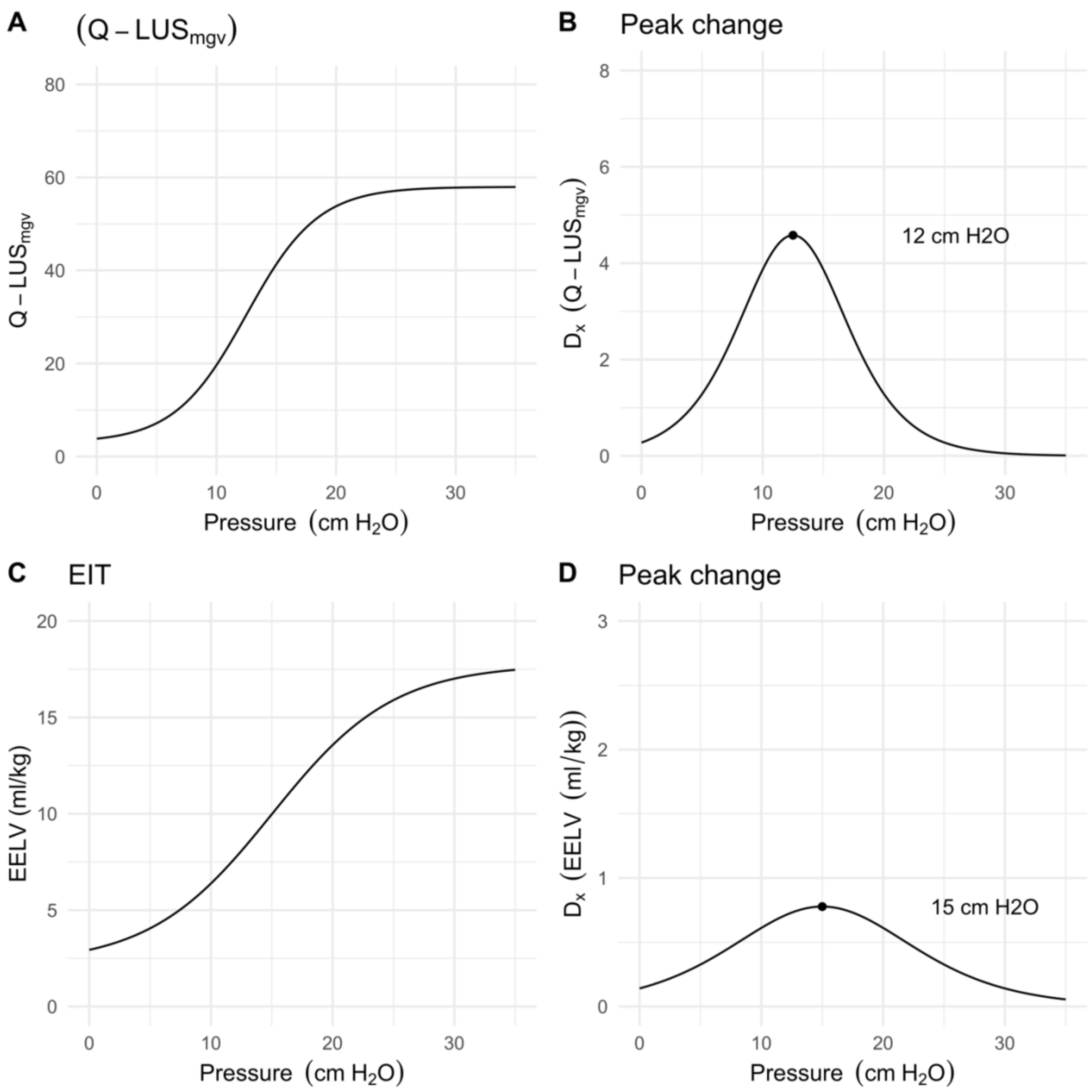

Supplementary Figure E14: Predicted closing pressure from fitting the mathematical model proposed by Venegas et al to inflation curves constructed from non-dependent Q-LUS$_{MGV}$ (A) and right whole lung EIT (C) respectively. Differential curves demonstrating peak change (black dot) in Q-LUS$_{MGV}$ (C) and EELV (D) are shown on the right. Closing pressure is at 12 cm H_2O and 15 cm H_2O from Q-LUS$_{MGV}$ and EIT respectively. EELV; End expiratory lung volume, Q-LUS$_{MGV}$; Quantitative lung ultrasound mean grey value. Pressure rounded to nearest whole number. Y axis scale adjusted per figure for ease of visualization.

1 Percie du Sert, N. et al. The Arrive Guidelines 2.0: Updated Guidelines for Reporting Animal Research. *PLOS Biology* **18**, e3000410 (2020).

2 Tingay, D. G. et al. Gradual Aeration at Birth Is More Lung Protective Than a Sustained Inflation in Preterm Lambs. *Am J Respir Crit Care Med* **200**, 608-616 (2019).

3 Blank, D. A. et al. Lung Ultrasound Immediately after Birth to Describe Normal Neonatal Transition: An Observational Study. *Arch Dis Child Fetal Neonatal Ed* **103**, F157-F162 (2018).

4 Hillman, N. H. et al. Brief, Large Tidal Volume Ventilation Initiates Lung Injury and a Systemic Response in Fetal Sheep. *Am J Respir Crit Care Med* **176**, 575-581 (2007).

5 Pereira-Fantini, P. M. et al. Preterm Lung Exhibits Distinct Spatiotemporal Proteome Expression at Initiation of Lung Injury. *Am J Respir Cell Mol Biol* **61**, 631-642 (2019).

6 Schindelin, J. et al. Fiji: An Open-Source Platform for Biological-Image Analysis. *Nat Methods* **9**, 676-682 (2012).

7 Alonso-Ojembarrena, A., Lechuga-Sancho, A. M., Ruiz-Gonzalez, E., Gonzalez-Haba-Martinez, B. & Lubian-Lopez, S. P. Pleural Line Thickness Reference Values for Preterm and Term Newborns. *Pediatr Pulmonol* **55**, 2296-2301 (2020).

8 Tingay, D. G. et al. Spatiotemporal Aeration and Lung Injury Patterns Are Influenced by the First Inflation Strategy at Birth. *Am J Respir Cell Mol Biol* **54**, 263-272 (2016).

9 Tingay, D. G. et al. Effectiveness of Individualized Lung Recruitment Strategies at Birth: An Experimental Study in Preterm Lambs. *Am J Physiol Lung Cell Mol Physiol* **312**, L32-L41 (2017).

10 Tingay, D. G. et al. Effect of Sustained Inflation Vs. Stepwise Peep Strategy at Birth on Gas Exchange and Lung Mechanics in Preterm Lambs. *Pediatr Res* **75**, 288-294 (2014).
